# Ice ages and butterflyfishes: Phylogenomics elucidates the ecological and evolutionary history of reef fishes in an endemism hotspot

**DOI:** 10.1002/ece3.4566

**Published:** 2018-10-23

**Authors:** Joseph D. DiBattista, Michael E. Alfaro, Laurie Sorenson, John H. Choat, Jean‐Paul A. Hobbs, Tane H. Sinclair‐Taylor, Luiz A. Rocha, Jonathan Chang, Osmar J. Luiz, Peter F. Cowman, Matt Friedman, Michael L. Berumen

**Affiliations:** ^1^ Red Sea Research Center, Division of Biological and Environmental Science and Engineering King Abdullah University of Science and Technology Thuwal Saudi Arabia; ^2^ Australian Museum Research Institute, Australian Museum Sydney New South Wales Australia; ^3^ School of Molecular and Life Sciences Curtin University Perth Western Australia Australia; ^4^ Department of Ecology and Evolutionary Biology University of California Los Angeles Los Angeles California; ^5^ College of Science and Engineering James Cook University Townsville Queensland Australia; ^6^ Section of Ichthyology California Academy of Sciences San Francisco California; ^7^ Research Institute for the Environment and Livelihoods, Charles Darwin University Darwin Northern Territory Australia; ^8^ ARC Centre of Excellence for Coral Reef Studies James Cook University Townsville Queensland Australia; ^9^ Department of Earth Sciences University of Oxford Oxford UK; ^10^ Museum of Paleontology and Department of Earth and Environmental Sciences University of Michigan Ann Arbor Michigan

**Keywords:** biogeographic barriers, *Chaetodon*, coral reef, glaciation events, Pleistocene, ultraconserved elements

## Abstract

For tropical marine species, hotspots of endemism occur in peripheral areas furthest from the center of diversity, but the evolutionary processes that lead to their origin remain elusive. We test several hypotheses related to the evolution of peripheral endemics by sequencing ultraconserved element (UCE) loci to produce a genome‐scale phylogeny of 47 butterflyfish species (family Chaetodontidae) that includes all shallow water butterflyfish from the coastal waters of the Arabian Peninsula (i.e., Red Sea to Arabian Gulf) and their close relatives. Bayesian tree building methods produced a well‐resolved phylogeny that elucidated the origins of butterflyfishes in this hotspots of endemism. We show that UCEs, often used to resolve deep evolutionary relationships, represent an important tool to assess the mechanisms underlying recently diverged taxa. Our analyses indicate that unique environmental conditions in the coastal waters of the Arabian Peninsula probably contributed to the formation of endemic butterflyfishes. Older endemic species are also associated with narrow versus broad depth ranges, suggesting that adaptation to deeper coral reefs in this region occurred only recently (<1.75 Ma). Even though deep reef environments were drastically reduced during the extreme low sea level stands of glacial ages, shallow reefs persisted, and as such there was no evidence supporting mass extirpation of fauna in this region.

## 1 INTRODUCTION

Explaining the underlying factors responsible for the diversity of species accumulated at hotspots of endemism remains a difficult problem in the field of biogeography. Recent research has identified the importance of peripheral regions from tropical oceans in generating and exporting biological diversity, thus intermittently seeding adjacent seas (Bowen, Rocha, Toonen, & Karl, 2013; DiBattista et al., 2013; DiBattista, Wilcox, Craig, Rocha, & Bowen, 2010; Eble et al., 2011; Gaither et al., 2011; Gaither, Toonen, Robertson, Planes, & Bowen, 2010; Malay & Paulay, 2010; Skillings, Bird, & Toonen, 2010); however, direct tests of this assumption are rare. Renewed interest in the Red Sea to Arabian Gulf (or Persian Gulf) region provides a new opportunity to explore hypotheses associated with how endemics are formed in peripheral areas, and its potential contribution to the species richness of marine biodiversity hotspots. The Red Sea is a semi‐enclosed basin located at the north‐western corner of the Indian Ocean and harbors one of the highest levels of endemism for marine organisms (12.9% for fishes, 12.6% for polychaetes, 8.1% for echinoderms, 16.5% for ascidians, and 5.8% for scleractinian corals; DiBattista, Roberts, et al., 2016). The level of endemism among well‐characterized groups in the Red Sea, such as the shore fishes, exceeds those of all other peripheral endemic hotspots identified for the Indian Ocean (DiBattista, Roberts, et al., 2016). Although many of these Red Sea endemics extend their distribution into the adjacent Gulf of Aden and Arabian Sea (DiBattista, Choat, et al., 2016; DiBattista, Roberts, et al., 2016; Kemp, 1998), it is not clear whether they are paleo‐endemics (old lineages restricted due to range contraction), neo‐endemics (young lineages at the site of origin), or “ecological” endemics (old or young lineages with a restricted range due to species ecology; see Cowman, Parravicini, Kulbicki, & Floeter, 2017) and where, when, and how this diversification occurred.

The Red Sea has a unique geological and paleoclimatic history that may have played a role in its high levels of endemism (see DiBattista, Choat, et al., 2016 for review). In brief, the Red Sea basin was formed by episodes of sea floor spreading 41–34 Ma (Girdler & Styles, 1974), followed by intermittent connections to the Mediterranean Sea in the north (~14–5 Ma; Hubert‐Ferrari et al., 2003), and a more recent connection to the Gulf of Aden in the south through the Strait of Bab al Mandab (~5 Ma to present; Bailey, 2010). The Strait is a narrow channel (29 km) with a shallow sill (137 m) that constitutes the only connection between the Red Sea and the Indian Ocean (Bailey, 2010). Water exchange is regulated by Indian Ocean monsoon patterns (Raitsos, Pradhan, Brewin, Stenchikov, & Hoteit, 2013; Smeed, 1997) but was historically minimal or absent during reduced sea levels caused by glacial periods of the Pleistocene (Rohling et al., 2009), including the most recent glacial maximum (20–15 ka; Ludt & Rocha, 2015; Siddall et al., 2003). Restricted water flow resulted in increased salinity within the Red Sea (Biton, Gildor, & Peltier, 2008), leading some to suggest that there was complete extirpation of Red Sea fauna during these periods (Klausewitz, 1989). The “Pleistocene extirpation” hypothesis, wherein all Red Sea fauna were eliminated during the last glacial maximum (~18 ka) and subsequently re‐populated via more recent colonization events, remains controversial and untested with modern comparative approaches (DiBattista, Choat, et al., 2016), although similar geological events may have occurred in the Mediterranean Sea (Bianchi et al., 2012). Thus, despite some agreement on the broad strokes of its geologic history, little consensus has emerged on the processes that shaped the Arabian Peninsula's present day marine biodiversity, their influence on biodiversity in adjacent regions, and the role of historical closures of the Strait of Bab al Mandab.

Butterflyfishes and bannerfishes, brightly colored reef fishes in the family Chaetodontidae, are a potential model system for elucidating the origins, maintenance, and evolutionary history of Red Sea endemics and their influence on species richness in adjacent marine regions. The family is diverse (17 species in the Red Sea and >130 species in the greater Indo‐West Pacific; Allen, Steene, & Allen, 1998) and phylogenetically well resolved compared to other reef fish families (Cowman, 2014). A high proportion of the Chaetodontidae species found in the coastal waters of the Arabian Peninsula are endemic (32%; DiBattista, Roberts, et al., 2016). Although recent molecular phylogenies of chaetodontids have helped to clarify many aspects of their evolutionary history (Bellwood et al., 2010; Cowman & Bellwood, 2011, 2013; Fessler & Westneat, 2007; Hodge, Herwerden, & Bellwood, 2014; Hsu, Chen, & Shao, 2007), a lack of sampling of Arabian Peninsula species has impeded our understanding of the diversification in this region.

The evolution of endemic species has been linked to ecological traits, such as reductions in dispersal ability and changes in body size (i.e., the island rule; reviewed by Lomolino, 2005; Whittaker and Fernández‐Palacios, 2007). For reef fishes, certain traits associated with dispersal ability are linked to geographic range size. For example, large, gregarious, and nocturnal species tend to have larger range sizes than small, solitary, and strictly diurnal species (Luiz et al., 2013, 2012). Moreover, dispersal ability can potentially influence clade diversification: to successfully colonize and establish populations in peripheral areas, tropical fish species must be good dispersers (Hobbs, Jones, Munday, Connolly, & Srinivasan, 2012). Following diversification in peripheral areas, newly formed lineages may evolve traits less conducive to dispersal, thus becoming endemic to the area where it originated, as often occurs in the evolution of insular terrestrial endemics (Whittaker and Fernández‐Palacios, 2007). We therefore predict that butterflyfishes endemic to the Arabian Peninsula region will have smaller body sizes, higher sociability, and reduced dispersal ability compared to their widespread congeners. Broadly speaking, endemic species tend to be ecological specialists and thus adapted to the environmental condition in which they arose (McKinney, 1997). We therefore additionally predict that these endemics will have a higher level of ecological specialization than widespread species. For reef fishes, habitat specialization is often defined by the depth range where they occur and the number of different habitats that they exploit (e.g., coral reefs, rocky reefs, seagrass beds, mangroves; Luiz et al., 2012). Dietary specialization is often defined by the proportion of different food categories targeted (Pratchett, 2014). We predict that butterflyfishes endemic to the Arabian Peninsula region will have higher dietary specialization and reliance on corals for food given recent origins alongside their coral rich habitat (Renema et al. 2016). We choose to focus on adult versus larval ecological traits because more information about the former is available, and has been shown to correlate with past (Ottimofiore et al., 2017) and present (Luiz et al., 2013) geographic range size.

The aims of this study are threefold. First, we aim to reconstruct the phylogeny and evolutionary timescale for Red Sea to Arabian Gulf butterflyfishes in order to test whether these peripheral areas intermittently seed the broader Indo‐West Pacific with biodiversity (“evolutionary incubator” hypothesis). Outcomes that would allow rejection of this hypothesis include a lack of evidence supporting Arabian Peninsular endemic fish lineages giving rise to Indo‐West Pacific fish lineages as well as restricted ancestral ranges expanding into this broader region. Second, we look to test the extent to which butterflyfish maintained a continuous presence in the Red Sea during the major environmental fluctuations of the Pleistocene (“Pleistocene extirpation” hypothesis). Outcomes that would allow rejection of this hypothesis include a lack of evidence supporting Arabian Peninsular endemic fish originating after the glacial cycles of the Pleistocene, as well as colonization events dated only before or after this epoch. Third, we aim to test whether species endemic to the coastal waters of the Arabian Peninsula non‐randomly associate with particular ecological traits (“ecological trait” hypothesis), which may be important in explaining patterns of diversification in this region. The expectation here is that endemic fishes are more specialized and thus better adapted to local conditions than their widespread congeners. Outcomes that would allow rejection of this hypothesis include a lack of association between endemism and any of the ecological traits considered here.

## 2 MATERIALS AND METHODS

### 2.1 Materials

Site location, sampling date, and museum voucher information (where available) for each specimen are outlined in Supporting Information Table S1. All butterflyfish species included in this study and their geographic distribution are listed in Table 1.

**Table 1:** Species distribution and clade designation from Bellwood et al. (2010) and Cowman and Bellwood (2011) for all Chaetodontidae samples used in this study

Species	Geographic distribution								
	Gulf of Aqaba (A)	Rest of Red Sea (B)	Djibouti and Gulf of Aden (C)	Socotra (D)	South Oman (E)	Arabian Gulf (F)	Gulf of Oman and Pakistan (G)	Rest of Indian Ocean (H)	Pacific Ocean (I)
Clade 4									
*Chaetodon auriga*	**√**	**√**	**√**	**√**	**√**		**√**	**√**	**√**
*Chaetodon auripes*									**√**
*Chaetodon collare*			**√**	**√**	**√**		**√**	**√**	**√**
*Chaetodon decussatus*			**√**					**√**	
Chaetodon dialeucos*			**√**		**√**				
*Chaetodon falcula*								**√**	
Chaetodon fasciatus*	**√**	**√**	**√**						
Chaetodon gardineri*			**√**	**√**	**√**		**√**	**√**	
*Chaetodon leucopleura*		**√**	**√**	**√**	**√**			**√**	
*Chaetodon lineolatus*	**√**	**√**	**√**	**√**				**√**	**√**
*Chaetodon lunula*			**√**	**√**	**√**			**√**	**√**
*Chaetodon melannotus*	**√**	**√**	**√**	**√**				**√**	**√**
Chaetodon mesoleucos*	**√**	**√**	**√**	**√**					
Chaetodon nigropunctatus*					**√**	**√**	**√**		
*Chaetodon oxycephalus*								**√**	**√**
Chaetodon pictus*		**√**	**√**	**√**	**√**		**√**		
Chaetodon semilarvatus*	**√**	**√**	**√**		**√**				
*Chaetodon vagabundus*				**√**				**√**	**√**
Clade 3									
Chaetodon austriacus*	**√**	**√**	**√**		**√**				
*Chaetodon baronessa*									**√**
*Chaetodon bennetti*				**√**				**√**	**√**
Chaetodon larvatus*	**√**	**√**	**√**		**√**				
*Chaetodon lunulatus*									**√**
Chaetodon melapterus*		**√**	**√**	**√**	**√**	**√**	**√**		
*Chaetodon plebeius*									**√**
*Chaetodon speculum*								**√**	**√**
*Chaetodon triangulum*								**√**	
*Chaetodon trifascialis*	**√**	**√**	**√**	**√**	**√**			**√**	**√**
*Chaetodon trifasciatus*			**√**	**√**				**√**	
*Chaetodon zanzibariensis*			**√**	**√**				**√**	
Clade 2									
*Chaetodon guttatissimus*				**√**				**√**	
*Chaetodon interruptus*								**√**	
*Chaetodon kleinii*				**√**				**√**	**√**
*Chaetodon madagaskariensis*								**√**	
*Chaetodon mertensii*									**√**
Chaetodon paucifasciatus*	**√**	**√**	**√**						
*Chaetodon pelewensis*									**√**
*Chaetodon punctatofasciatus*								**√**	**√**
*Chaetodon trichrous*									**√**
*Chaetodon unimaculatus*								**√**	**√**
*Chaetodon xanthurus*									**√**
Bannerfishes									
*Forcipiger flavissimus*		**√**	**√**	**√**				**√**	**√**
*Forcipiger longirostris*				**√**				**√**	**√**
*Heniochus acuminatus*			**√**	**√**	**√**	**√**	**√**	**√**	**√**
*Heniochus diphreutes*	**√**	**√**	**√**	**√**				**√**	**√**
Heniochus intermedius*	**√**	**√**	**√**						

**Figure 1:**
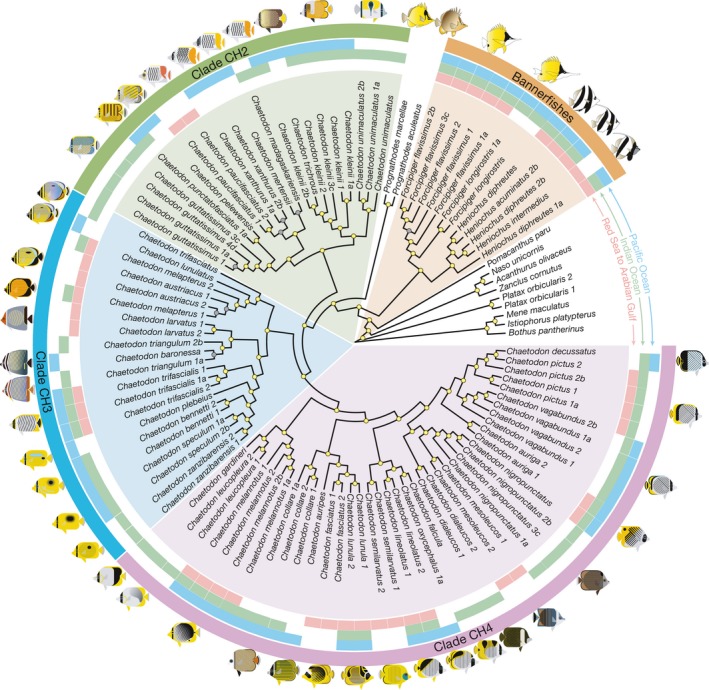
Inferred phylogeny of Red Sea to Arabian Gulf butterflyfish species, including some of closest their congeners, based on ExaBayes analysis of ultraconserved element data. Yellow dots on node labels indicate a posterior probability of 1, whereas gray dots indicate a posterior probability of <1 but >0.6. Clades based on Bellwood et al. (2010) and Cowman and Bellwood (2011) are indicated. Records for each species are mapped onto the topology as follows: red = Red Sea to Arabian Gulf, green = rest of Indian Ocean, and blue = Pacific Ocean

Colors in the table header match the colors used to denote species distributions in Figure 1. Asterisks indicate regional endemics for the purposes of our correlational trait analysis. The letters below each region indicate the geographic groupings used for BioGeoBEARS analysis. Although *Chaetodon leucopleura*, *Chaetodon melapturus*, and *Chaetodon pictus* are listed as being present in the Red Sea, this is based on rare records at their northern limits. Similarly, we have only sampled *C. pictus* (and not *Chaetodon vagabundus*) at Socotra (DiBattista et al., 2017), and rare records of *Chaetodon austriacus* in the Gulf of Aden and South Oman likely represent waifs.

As our primary objective is to reconstruct the evolutionary history of butterflyfishes known to occur in the Red Sea and adjacent gulfs or seas, we concentrated our sampling efforts on those species and their closest relatives. Although five major Chaetodontidae lineages were sampled, Chaetodon Clade CH1 (*Chaetodon robustus* and *C. hoefleri*, restricted to the Atlantic; Cowman & Bellwood, 2013), and multiple bannerfish genera (*Amphichaetodon*,* Chelmon*,* Chelmonops*,* Coradion*,* Hemitaurichthys*, and *Johnrandallia*) without species represented in the Red Sea were not sampled in this study. Two species of the *Prognathodes* genus were included to facilitate fossil calibration, but were not included in the biogeographic analyses due to their Atlantic distributions (see below).

In total, we sampled 47 chaetodontid species (35% of the entire family), which includes all regional endemics and wide‐ranging species found in the Arabian Peninsula region save *Roa jayakari*, a rare deepwater species distributed from the Red Sea to coastal India; we were unable to secure a tissue sample as part of this study. Eight of these species have not previously been sampled in phylogenetic studies of the family (Bellwood et al., 2010; Cowman & Bellwood, 2011; Fessler & Westneat, 2007; Hodge et al., 2014). Tissues were preserved in a saturated salt‐DMSO solution or 95% ethanol prior to processing. This research was carried out under the general auspices of King Abdullah University of Science and Technology's (KAUST) arrangements for marine research with the Saudi Arabian Coast Guard and the Presidency of Meteorology and Environment. The animal use protocol was approved by KAUST's Biosafety and Ethics Committee (KAUST does not provide specific approval number).

### 2.2 Phylogenomics approach

We employ the sequence capture method of ultraconserved elements (UCEs) to produce millions of reads in parallel from multiple butterflyfish specimens collected from the Gulf of Aqaba in the west (Red Sea) to the Hawaiian Archipelago in the east (Pacific Ocean, PO). UCEs are a class of highly conserved and abundant nuclear markers distributed throughout the genomes of most organisms (Bejerano, Haussler, & Blanchette, 2004; Siepel et al., 2005; Reneker et al., 2012). These markers do not intersect paralogous genes (Derti, Roth, Church, & Wu, 2006), do not have retro‐element insertions (Simons, Pheasant, Makunin, & Mattick, 2006), have a range of variant sites (i.e., evolving on different time scales; Faircloth et al., 2012), and have been used to reconstruct phylogenies across vertebrates (Bejerano et al., 2004; Faircloth et al., 2012; Faircloth, Sorenson, Santini, & Alfaro, 2013; McCormack et al., 2013; Smith et al., 2014; Sun et al., 2014), including fishes at both shallow (Mcgee et al., 2016) and deep (Alfaro et al., 2018; Faircloth et al., 2013; Harrington et al., 2016) phylogenetic scales.

### 2.3 DNA library preparation and next‐generation sequencing

DNA was extracted with DNeasy Blood and Tissue kits (Qiagen, Valencia, CA), which included an RNAse A treatment step. Each extracted sample was visualized by gel electrophoresis to assess DNA quality. Total DNA from each extracted aliquot was quantified using a Qubit dsDNA HS Assay Kit (Invitrogen, Carlsbad, CA), and 1.2 µg of DNA per individual sample was fragmented by sonication to 500 base pairs (bp) using a Covaris S2 sonicator (Covaris Inc, Woburn, MA) and used for UCE library preparation. In brief, we end‐repaired, adenylated, and ligated fragmented DNA to Illumina TruSeq‐style adapters, which included custom sequence tags to barcode each individual sample (Faircloth & Glenn, 2012). Following an 18‐cycle PCR to amplify indexed libraries for enrichment, we created pools by combining 62.5 ng of eight individual libraries. Each pool was concentrated to 147 ng/μl using a vacuum centrifuge. We then followed an established workflow for target enrichment (Gnirke et al. 2009) with modifications specified in Faircloth et al. (2012). Specifically, we enriched each pool, targeting UCE loci and their flanking sequence, using synthetic RNA capture probes (MyBaits, Mycroarray, Inc., Ann Arbor, MI). We combined the enriched, indexed pools at equimolar ratios prior to sequencing. The two final pooled libraries were each run paired‐end (150 bp sequencing) on independent lanes of an Illumina HiSeq2000 (v3 reagents) at the KAUST Bioscience Core Lab. Detailed methods of library enrichment, post‐enrichment PCR, and validation using relative qPCR may be found at https://ultraconserved.org/#protocols.

### 2.4 Sequence read quality control, assembly, and UCE identification

We removed adapter contamination and low quality bases with illumiprocessor (Faircloth, 2013), a parallel wrapper to Trimmomatic (Bolger, Lohse, & Usadel, 2014). To assemble the trimmed dataset, we used the PHYLUCE pipeline (version 8ca5884; Faircloth, 2016) with the phyluce_assembly_assemblo_trinity.py wrapper script for Trinity (version 1.5.0; Grabherr et al., 2011). We matched assembled contigs to enriched UCE loci by aligning contigs from each species to our UCE probes using the phyluce_assembly_match_contigs_to_probes.py script with the LASTZ assembler (Harris, 2007). We stored these match results into a SQLite relational database after excluding contigs that matched multiple UCE loci and UCE loci whose probes matched multiple contigs.

We used phyluce_align_seqcap.py to align UCE loci with MAFFT (Katoh & Standley, 2013; Katoh, Misawa, Kuma, & Miyata, 2002). Following alignment, we end‐ and internally‐trimmed alignments with GBLOCKS (Castresana, 2000) to improve phylogenetic inference by removing poorly aligned or highly divergent sites (Talavera & Castresana, 2007). We selected loci that were present in at least 75% of our specimens and concatenated the alignments into a PHYLIP‐formatted matrix for phylogenetic analysis. We included previously published UCE data for three species in our alignment to represent Acanthomorpha outgroup lineages and more accurately calibrate the phylogeny (see below).

### 2.5 Phylogenetic analysis of concatenated UCE data: evaluation of the “evolutionary incubator” and “Pleistocene extirpation” hypotheses

We fully partitioned our concatenated alignment by UCE locus and performed Bayesian analyses of the dataset with ExaBayes (Aberer, Kobert, & Stamatakis, 2014) and two independent runs, sampling every 500 generations. We used the autostopping convergence criteria of an average standard deviation of split frequencies of <5% and visualized the log‐likelihood of each chain to ensure convergence in Tracer version 1.6 (Rambaut et al., 2014).

We estimated divergence times using MCMCTREE in the PAML package on the Bayesian consensus topology. We used the likelihood approximation approach following the two‐step procedure described by Dos Reis and Yang (2011) by first estimating a mean substitution rate for the entire alignment with BASEML under a strict molecular clock and then using this estimate to set the rgene_prior in MCMCTREE. We used a single, unpartitioned alignment for computational tractability, with an HKY85 model, five categories for the gamma distribution of rate heterogeneity, an rgene_gamma prior for the gamma distribution describing gene rate heterogeneity of (2, 371.0575, 1) and a sigma2_gamma prior of (2, 5, 1). We adopted a calibration strategy that builds on Harrington et al. (2016) by including more proximal acanthomorph outgroups to Chaetodontidae and their immediate relatives. We constrained six nodes on the basis of fossil information using hard lower and soft upper bounds outlined in Supporting Information Figure S1. We assigned a minimum amount of prior weight for ages below the lower bound (1e‐200) and 5% prior weight for ages higher than the upper bound. Briefly, we link a series of carangimorph, syngnathiform, holocentroid, and lampridiform fossils to the sequences of acanthomorph outgroup fossils as per Harrington et al. (2016). This resulted in the following outgroup node calibrations: acanthuroids versus all other taxa (lower bound: 54.17 Ma; upper bound: 70.84 Ma); acanthurids versus zanclids (lower bound: 49.0 Ma; upper bound: 62.7 Ma), *Naso* versus *Acanthurus* (lower bound: 49.0 Ma; upper bound: 57.22 Ma), Chaetodontidae versus Pomacanthidae (lower bound: 29.62 Ma; upper bound: 59.26 Ma), and the total‐group *Chaetodon* versus *Prognathodes* (lower bound: 7 Ma; upper bound: 47.5 Ma). Further justification for calibrations is available as Supporting Information (Appendix S1).

### 2.6 Ancestral biogeographic range estimation: evaluation of the “evolutionary incubator” and “Pleistocene extirpation” hypotheses

We estimated ancestral distribution patterns for chaetodontid lineages using the pruned time‐calibrated phylogeny analyzed with the R package BioGeoBEARS (Matzke, 2013), which allows several models of biogeographic evolution to be compared via likelihood inference, and the ability to incorporate a parameter allowing for founder‐event speciation. For these analyses, we coded each taxon based on presence/absence in nine discrete geographical areas: Gulf of Aqaba, rest of the Red Sea, Djibouti and Gulf of Aden, Socotra, South Oman, Arabian Gulf, Gulf of Oman and Pakistan, rest of Indian Ocean, and PO. The discrete coding of geographic areas adjacent to the Arabian Peninsula enables a fine‐scale investigation of the ancestral biogeography of that region for our taxa of interest. Presence/absence and geographical range data for each taxon were obtained from a combination of DiBattista, Roberts, et al. (2016) and FishBase (Froese & Pauly, 2011). *Prognathodes* spp. (a Chaetodontidae genus) were not considered in this part of the analysis given that these two taxa are restricted to tropical Atlantic waters.

We constrained our biogeographic analyses to prohibit colonization events between the Red Sea and Indian/PO regions before 5 Ma reflecting the time when a more permanent connection was formed via the Strait of Bab al Mandab (Bailey, 2010). Our BioGeoBEARS analysis evaluated the DEC, DIVALIKE, and BAYAREALIKE models with and without the jump (*J*) parameter (Matzke, 2013). These models describe biogeographic scenarios where dispersal, extinction, cladogenesis, vicariance, and founder events are differentially invoked to explain present day distributional patterns. In our case, we were interested in whether the range‐restricted endemics from the coastal waters of the Arabian Peninsula represent ancient relicts, new colonization events, and/or a source of biodiversity (at some point in the past) for the broader Indo‐West Pacific.

### 2.7 Comparative trait analysis: evaluation of the “ecological trait” hypothesis

In order to determine whether particular species‐level traits were associated with the evolution of endemism in this subset of Chaetodontidae species, we fitted a phylogenetic generalized linear model (function “phyloglm” in R package “phylolm” [Ho et al., 2016]) that assumed “regional endemism” (i.e., endemic to the coastal waters of the Arabian Peninsula; DiBattista, Roberts, et al., 2016) as the binomial response variable and a suite of ecological traits as the predictive fixed factors. For model selection, we performed a backward stepwise procedure for PGLM's (function “phylostep” in R package “phylolm” [Ho et al., 2016]), which entailed sequential optimization by removing non‐influential fixed‐effect terms from the full model based on Akaike information criteria (AIC). Full details on the methods and data sources are provided in Supporting Information Table S2. We also explore interactions among the predictive traits using a regression tree approach (De’ath and Fabricius, 2000; function “rpart” in R package “rpart” [Therneau et al., 2015]).

Among the predictive variables considered were: maximum body size (total length = TL; Allen et al., 1998; Kuiter, 2002), depth range inhabited (Allen et al., 1998), social structure (three categories ordered from low to high sociability: solitary, pair formation, and group formation; Allen et al., 1998; Kuiter, 2002; Yabuta and Berumen, 2013), habitat breadth (estimated as the sum value of all habitat types inhabited: C = coral, R = rocky, D = deep reef, S = sediment, R = rubble, CO = coastal, CA = algal beds; Allen et al., 1998; Kuiter, 2002), and dietary reliance on coral reefs (four categories ordered from low to high reliance: planktivore, benthic invertivore, facultative corallivore, and obligate corallivore; Cole and Pratchett, 2014). We also included the phylogenetic age of species (Myr) as an additional fixed factor to test whether species traits are influenced by time of divergence from sister taxa. For phylogenetic age, we evaluate for each species (regional endemic and widespread) whether we sampled its closest sister species by comparing our phylogeny with those published previously (Cowman & Bellwood, 2011) and other published accounts (Kuiter, 2002). The ecological traits were selected because they are associated with specialization, fitness, and range expansion in butterflyfishes, and thus may help to explain patterns of evolution in fish endemic to the coral reefs of the Arabian Peninsula. We do note this may be an oversimplification given that our categories are coarse and biased toward adult versus larval traits, which are themselves data deficient. Previous work, however, has demonstrated that traits associated with the successful recruitment of reef fish are more important than traits associated with dispersal in determining differentiation between habitats (Gaither et al., 2015; Keith, Woolsey, Madin, Byrne, & Baird, 2015).

## 3 RESULTS

### 3.1 UCE sequences

Reads, contigs, and UCE loci per individual are outlined in Supporting Information Table S3. In summary, we sequenced a total of 153.31 million reads, with a mean of 1.55 million reads per sample from 47 focal taxa (excluding outgroups; also see Table 1). Overall, we assembled a mean of 12,969 contigs (95 CI, min = 10,593, max = 15,345) and 901 UCE loci per sample (95 CI, min = 871, max = 932).

### 3.2 Phylogenetic reconstruction and timing of divergence: evaluation of the “evolutionary incubator” and “Pleistocene extirpation” hypotheses

Following assembly, alignment, trimming, and filtering out loci that were present in fewer than 75 specimens (for a 75% complete dataset), we retained 971 alignments with a mean length of 515.6 bp. The concatenated supermatrix contained 500,642 bp with 52,680 informative sites and was 83.3% complete based on the proportion of non‐gap sequences. The following samples were excluded from further analysis due to the low number of loci recovered: *Chaetodon_interruptus*1a, *Chaetodon_lineolatus*1a, *Chaetodon_lunula*1a, and *Chaetodon ulietensis*1a (for full details see Supporting Information Table S1); however, tissue replicates were retained for two of the four species listed here (*Chaetodon lineolatus* and *Chaetodon lunula*).

**Figure 2:**
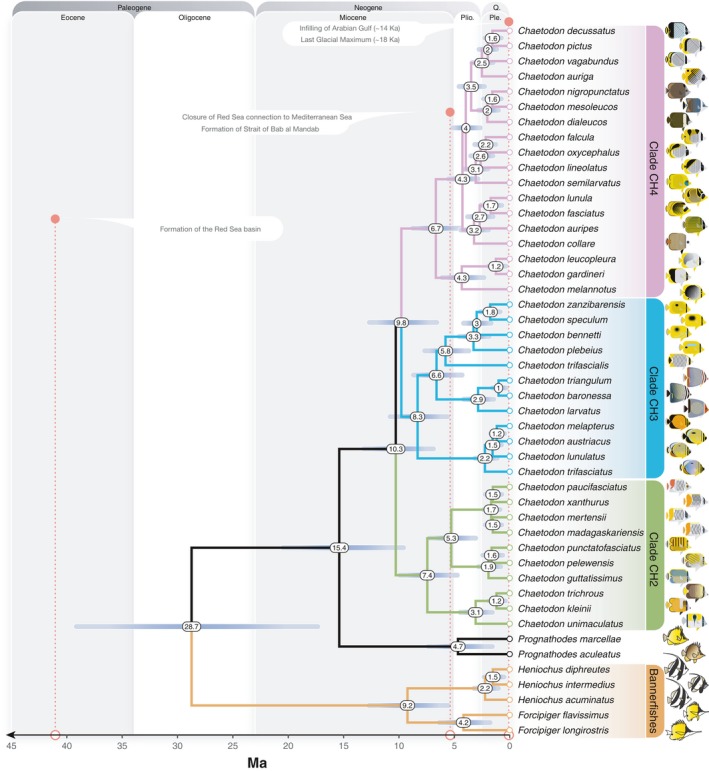
A fossil calibrated chronogram for select Chaetodontidae species based on analysis of ultraconserved element data. The time scale is calibrated in millions of years before present. Node ages are presented as median node heights with 95% highest posterior density intervals represented by bars. Significant geological events in the coastal waters of the Arabian Peninsula are temporally indicated by red dashed lines

Our Bayesian and maximum likelihood analyses produced a fully resolved topology that shared key points of congruence with prior multi‐locus studies of butterflyfishes (Bellwood et al., 2010; Cowman & Bellwood, 2011; Fessler & Westneat, 2007; Hodge et al., 2014 ; Hsu et al., 2007; see Supporting Information Figure S2). Although direct comparisons to previous phylogenies are difficult because these are missing many of the regional endemics (e.g., *Chaetodon dialeucos*, *C. gardineri*, *C. leucopleura*, *C. nigropunctatus*, *C. pictus*, *C. triangulum*, *Heniochus intermedius*), and contain less sequence data and data overlap (e.g., six loci and 73% complete matrix; Hodge et al., 2014), where there was overlap in the data sets the tips of the tree displayed similar topologies (Supporting Information Figure S3). In our case, however, almost every node in the tree was strongly supported (posterior probabilities of 1.0; Figures 1 and 2).

**Figure 3:**
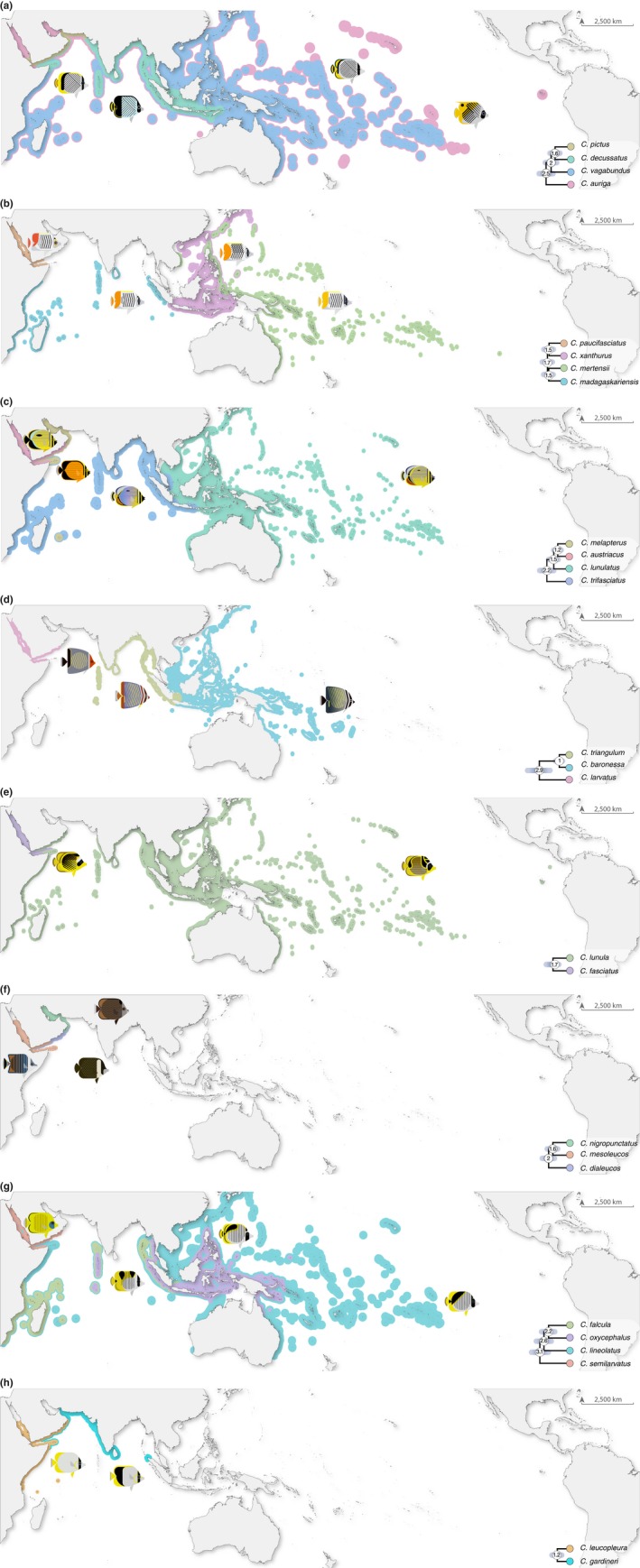
Distributions, range overlap, and ages of divergence in eight clades of butterflyfish from the *Chaetodon* genus that contain species inhabiting the Red Sea to Arabian Gulf region. Clade structure and node ages (median node heights with 95% highest posterior density intervals represented by bars) were extracted from Figure 2

By only considering a single representative sample per species on our chronogram (Figure 2), we found that the majority of Red Sea to Arabian Gulf butterflyfish diverged from their closest relatives in the last five million years (4.17–1.16 Ma), with an average lineage age of 2.39 Ma. In comparison to previous fossil calibrated studies of Chaetodontidae (Cowman & Bellwood, 2011; Hodge et al., 2014), the mean ages and 95% highest posterior density (HPD) estimates are more restricted, but for the most part overlap with previous estimates (Supporting Information Figure S3). In terms of the topology, although our phylogenetic sampling is restricted, it still captures crown nodes and age estimates of major chaetodontid lineages (with the exception of the bannerfish lineage), as well as subclades containing Red Sea to Arabian Gulf species and their most recent common ancestors (Supporting Information Figure S2). Most of the clades included species pairs diverging with non‐overlapping distributions dating back 2–1 Ma. This divergence does not appear to coincide with the timing of the emergence of apparent geographic (and geological) barriers such as the Strait of Bab al Mandab (Figures 2 and 3). Regional endemics appear to have diverged earliest from ancestors that gave rise to the clades including *Chaetodon larvatus* and *Chaetodon semilarvatus*. At least one entire subclade of CH4 was comprised of regional endemics (*C. dialeucos*, *C. nigropunctatus*, and *C. mesoleucos*). The split between the butterflyfishes (*Chaetodon*, *Prognathodes*) and bannerfishes (*Heniochus*, *Forcipiger*) was much older, with a mean of 28.7 Ma (95% HPD: 40.0–18.26; Figure 2 and Supporting Information Figure S1), indicating an ancient split between these highly divergent body forms.

### 3.3 Ancestral range reconstruction: evaluation of the “evolutionary incubator” and “Pleistocene extirpation” hypotheses

**Figure 4:**
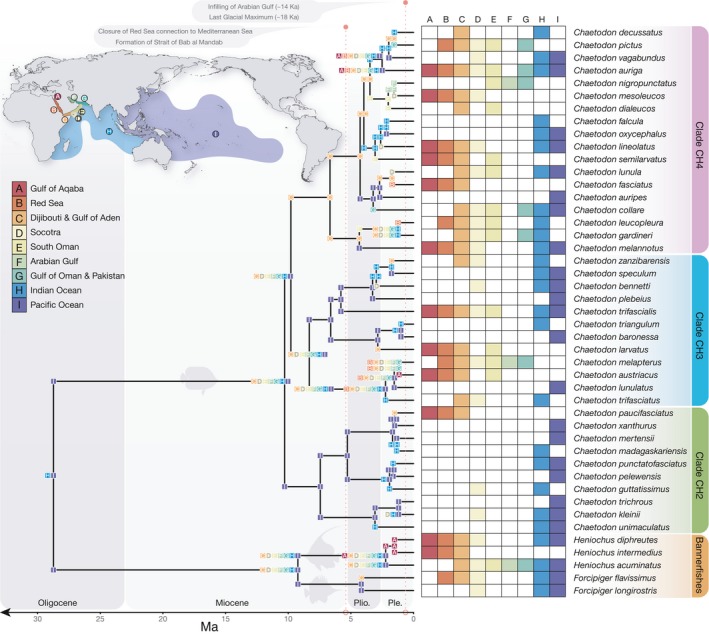
Ancestral range estimations inferred using the DEC+J model based on a time‐calibrated Bayesian phylogeny of Chaetodontidae species. States at branch tips indicate the current geographical distributions of taxa, whereas states at nodes indicate the inferred ancestral distributions before speciation (middle) and after (corner). The regions considered in this analysis include the following: Gulf of Aqaba, rest of Red Sea, Djibouti and Gulf of Aden, Socotra, South Oman, Arabian Gulf, Gulf of Oman and Pakistan, rest of Indian Ocean, and Pacific Ocean. Abbreviations: Plio. = Pliocene; Ple. = Pleistocene. Significant vicariance in the Red Sea to Arabian Gulf region is indicated by red dashed lines

Model comparison revealed the DEC+J model as the most likely (LnL = −250.79, AIC weight = 0.76) and the DIVALIKE+J model as the second most likely (LnL = −252.76, AIC weight = 0.11; Table 2 and Figure 4). The importance of the *J* parameter, which models long‐distance or “jump” dispersal, is that ancestral ranges often comprise one area rather than several areas. The addition of the *J* parameter resulted in a significantly better model fit for DEC models when compared via a likelihood ratio test (LRT: *D *=* *8.67, *p *=* *0.0032).

**Table 2:** Akaike information criterion (AIC) model testing based on distribution patterns for butterflyfish lineages using the time‐calibrated phylogeny analyzed with the R module BioGeoBEARS, where *d* represents the dispersal parameter, *e* represents the extinction parameter, and *j* represents founder‐event speciation

	Ln likelihood	Number of parameters	*d*	*e*	*j*	AIC	AIC weight
DEC	−255.13	2	0.06	0	0	514.25	0.03
**DEC+J**	**−250.79**	**3**	**0.05**	**0**	**0.04**	**507.58**	**0.76**
DIVALIKE	−253.88	2	0.07	0.04	0	511.76	0.09
DIVALIKE+J	−252.76	3	0.06	0.02	0.03	511.52	0.11
BAYAREALIKE	−259.86	2	0.05	0.18	0	523.71	0
BAYAREALIKE+J	−255.48	3	0.04	0.08	0.06	516.96	0.01

For these models, we coded each taxon based on presence/absence in nine discrete geographical areas: (A) Gulf of Aqaba, (B) rest of Red Sea, (C) Djibouti and Gulf of Aden, (D) Socotra, (E) South Oman, (F) Arabian Gulf, (G) Gulf of Oman and Pakistan, (H) rest of Indian Ocean, and (I) Pacific Ocean. Bold indicates the favored model based on AIC scores.

Under the DEC+J model, Chaetodontidae had their crown origins in the Indo‐West Pacific, with subsequent dispersal to include the Arabian Peninsula and lineages leading to the base of *Chaetodon* and the bannerfish clade (*Forcipiger/Heniochus*; Figure 4). Within the CH2 clade, diversification was restricted to the PO with subsequent dispersal to the Indian Ocean (*Chaetodon madagaskariensis*,* C. punctatofasciaticus*,* and C. unimaculatus*), and three of the species have dispersed as far as Socotra (*Chaetodon guttatissimus*,* C. kleinii*,* and C. trifasciatus*). Only one species within CH2 diverged in the Gulf of Aden and subsequently colonized the Red Sea (*Chaetodon paucifasciatus*). The age of *C. paucifasciatus* is relatively young (1.5 Ma, HPD: 0.8–2.3 Ma), suggesting a similar timeline for its occupation of the Red Sea from the Gulf of Aden.

In the CH3 clade, three species were present in the Red Sea that were also restricted to the Arabian Peninsula (*Chaetodon austriacus*,* C. melapterus*,* and C. larvatus*). In the case of sister pair *C. austriacus* and *C. melapterus*, the reconstruction suggests that speciation occurred by vicariance within the Red Sea. Although posterior probabilities make the details of this split equivocal, the most likely scenario is a split between the Gulf of Aqaba and the Red Sea, where *C. austriacus* subsequently recolonized the entire Red Sea but *C. melapterus* expanded out to the Gulf of Aden, Arabian Sea, and Arabian Gulf, but no further. The extended history of the clade, although not completely sampled (see Supporting Information Figure S2), suggests that a widespread ancestor expanded into the Red Sea with subsequent vicariance between the PO, Indian Ocean, and Arabian Peninsula sites. Indeed, *C. larvatus* appears to originate in Djibouti and the Gulf of Aden followed by dispersal into the Red Sea and South Oman. *Chaetodon trifascialis*, on the other hand, maintained connections across the Indo‐West Pacific with subsequent range expansion into the Red Sea.

The CH4 clade has been the most successful in terms of butterflyfish colonizing the Red Sea. Eight extant species from CH4 are distributed in at least some parts of the Red Sea (*Chaetodon auriga*, *C. fasciatus*, *C. leucopleura*, *C. lineolatus*, *C*. *melannotus*,* C. mesoleucos*,* C. pictus*,* and C. semilarvatus)*, four of which are restricted to the Arabian Peninsula (*C. fasciatus*,* C. mesoleucos*,* C. pictus*,* and C. semilarvatus*). Moreover, the reconstruction identified a mix of origin states for CH4 species found in the Red Sea. Both *C. fasciatus* and *C. leucopleura* have their origins within the Red Sea, whereas *C*. *lineolatus* and *C. mesoleucos* have their origins at Socotra. The origins of *C. semilarvatus* are placed in South Oman, whereas the origins of *C. pictus* are placed in the Gulf of Oman. With the exception of *C. lineolatus*, a widespread Indo‐West Pacific species, all CH4 lineages have origins in the Arabian Peninsula region and Indian Ocean, and subsequent dispersal was limited from these sites. *Chaetodon lineolatus* appears to be the only species in CH4 to originate in the Arabian Peninsula and then disperse across the broader Indo‐West Pacific. However, unsampled taxa from this clade are more widely distributed across the Indian and POs (Supporting Information Figure S2).

Three taxa of the bannerfish clade are also present in the Red Sea (*Heniochus diphreutes, H. intermedius*,* Forcipiger flavissimus*), with *H. intermedius* considered a Red Sea to Gulf of Aden endemic. Despite these taxa only being representative of a small proportion of the entire bannerfish clade, the reconstruction suggests a widespread ancestor that diverged in the Arabian Peninsula region (*H. intermedius*) with subsequent (successful) colonization of the broader Indo‐West Pacific (*H. diphreutes* and *F. flavissimus*).

### 3.4 Correlational trait analysis: evaluation of the “ecological trait” hypothesis

**Figure 5:**
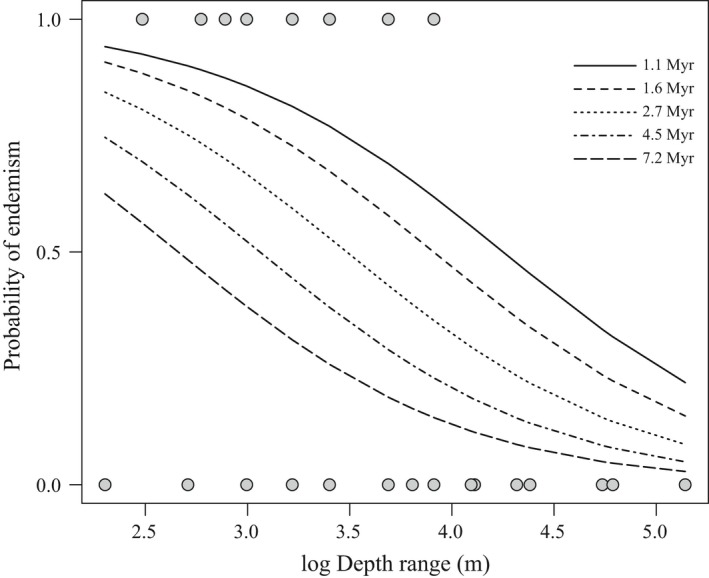
Estimated probability of endemism among Red Sea to Arabian Gulf butterflyfish species, including some of their closest congeners, as a function of depth range. Different line types represent variability in estimated species phylogenetic age extracted from Figure 2 (see legend)

**Figure 6:**
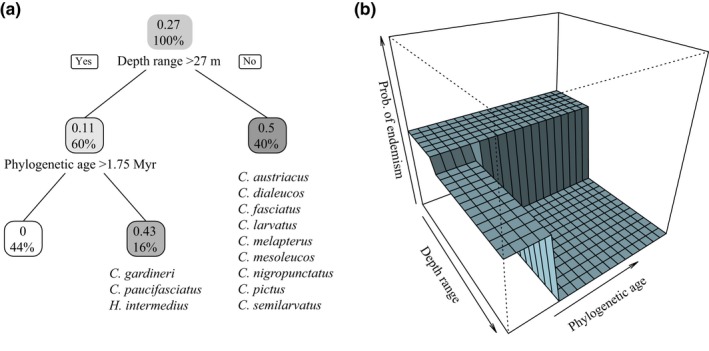
The classification of species‐level traits associated with endemism among the Red Sea to Arabian Gulf butterflyfishes (a). Data on the leaves (represented by squares) provide the probability of endemism (top) and the percentage of all observations in the node (bottom). The right panel shows the prediction surface (b)

Based on the best‐fit PGLM, depth range and phylogenetic age were negatively correlated with endemism, with depth range being a stronger predictor than phylogenetic age (Table 3, Figures 5 and 6). Exploring these relationships using a regression tree approach reveals that the effect of phylogenetic age is dependent on depth range. Endemic species from the Arabian Peninsula region are therefore more likely to be younger than widespread ones, but only for those species with depth ranges extending to mesophotic reefs (depth range >27 m; Figures 5 and 6). Endemism was not correlated with any of the other factors in the analysis for the butterflyfishes considered here (Supporting Information Tables S2 and S4).

**Table 3:** Summary of the final (best) phylogenetic, linear multi‐regression model, based on estimated probability of endemism as a response variable, selected after the backward stepwise phylostep procedure

	Estimate	*SE*	*z* value	*p* value
(Intercept)	6.170	2.506	2.461	**0.013**
Depth range	−1.423	0.543	−2.620	**0.008**
Phylogenetic age	−1.209	0.694	−1.742	0.061

Coefficients in bold indicate significance (*p* **<* *0.05).

## 4 DISCUSSION

This study used 901 loci to successfully generate a genome‐scale phylogeny of bannerfishes and butterflyfishes endemic to the coastal reefs of the Arabian Peninsula. This is the first time this genomic method has been applied to species‐level phylogenetic analyses of a reef fish group. Our phylogeny, which includes all shallow water chaetodontid species found in the Red Sea to Arabian Gulf and their close relatives distributed throughout the Indo‐West Pacific, provides divergence times with narrow confidence intervals and biogeographic insight into this endemism hotspot. Reconstructing the evolutionary history of these fishes with their widespread relatives does not appear to support the evolutionary incubator hypothesis. That is, despite generating significant biodiversity in the form of endemic species, these peripheral areas of the Arabian Peninsula do not appear to have exported significant biodiversity to the central Indo‐West Pacific. In fact, potentially only three species with reconstructed origins in the Arabian Peninsula (*C. lineolatus*, *H. diphreutes*, and *F. flavissimus*) appear to subsequently disperse to the Indo‐West Pacific. Our phylogenetic analyses also revealed that most endemic species originated prior to and persisted through the major environmental fluctuations of the Pleistocene, which does not support the Pleistocene extirpation hypothesis. The ecological trait‐based analyses revealed that the evolution of Red Sea to Arabian Gulf endemic butterflyfishes was associated with specialization to shallow reef habitat and, to a lesser extent, species’ phylogenetic age.

### 4.1 Evaluation of the “evolutionary incubator” hypothesis

The Red Sea, Gulf of Aden, Arabian Sea, and Arabian Gulf are all peripheral to the broader Indo‐West Pacific biogeographic region and potentially produce/contribute new reef fish species to the center (see Bowen et al., 2013; Hodge et al., 2014). Temporally, the Red Sea to Arabian Gulf butterflyfish assemblage (17 species in total) is made up of recently diverged lineages, with ages ranging from 4.17 Ma (*F. flavissimus*) to 1.16 Ma (*C. austriacus/C. melapterus* split). In a few cases, the Red Sea to Gulf of Aden endemics appear to have diverged as the earliest lineage of that clade (e.g., *C. larvatus* and *C. semilarvatus*; Figures 2 and 3). Indeed, the “oldest” endemic butterflyfish lineage in our study, *C. larvatus* (2.86 Ma, 4.3–1.6 Ma 95% HPD), appeared in the late Pliocene, and diverged from an Indo‐West Pacific lineage that later gave rise to species allopatric between the two ocean basins (*C. triangulum* in the Indian Ocean and *C. baronessa* in the PO). The ancestral range reconstruction of these Arabian Peninsula endemics demonstrates consistent colonization routes to the Red Sea and Arabian Sea via the Indian Ocean from the east (Figure 4), but with few examples of reciprocal expansion from the Arabian Peninsula back to the Indian Ocean and PO. For example, both *C. larvatus* and *C. semilarvatus* appear to have historically diverged in Djibouti/Gulf of Aden and South Oman, respectively, successfully colonized the Red Sea, but not established further south and east based on present day distributions. Similar reconstruction results were obtained for the regional endemic *C. pictus* (Red Sea to Gulf of Oman), which showed apparent historical divergence in the Gulf of Oman and only recent colonization of the southern limits of the Red Sea.

Other endemics appear to have historically diverged within the Red Sea (*C. fasciatus*) or adjacent Djibouti and Gulf of Aden (*C. paucifasciatus*) but not colonized any further to the southeast. Although equivocal based on the probabilistic uncertainty of nodes in the ancestral range reconstruction of the most likely model (DEC+J), there are a number of competing explanations for how *C. austriacus* and *C. melapterus* diverged from each other within the coastal waters of the Arabian Peninsula (also see Waldrop et al. 2016), particularly since *C. melapterus* is the only species in this complex present in the Arabian Gulf. The most likely explanation is based on present day distributions (Figure 3c): *C. austriacus* is largely restricted to the northern and central Red Sea (with rare records in the southern Red Sea and outside of the Red Sea), whereas *C. melapterus* is most abundant within or adjacent to the Arabian Gulf (with rare records in the southern Red Sea)—these bodies of water show opposite trends in terms of productivity, sea surface temperature, and temporal patterns of environmental variation (Pous, Lazure, & Carton, 2015; Raitsos et al., 2013). These environmental conditions are additionally significantly different from the rest of the Indian Ocean, and thus, the unique conditions in the Red Sea and Arabian Gulf may help explain how endemics evolved, or at least, concentrated and persisted in these peripheral locations.

Despite a lack of supporting evidence for the evolutionary incubator hypothesis, a clear pattern emerges that the unique environmental conditions in these peripheral seas may have contributed to the formation of endemic species as outlined above. For example, some butterflyfish subclades are comprised entirely of regional endemics (e.g., *C. dialeucos*,* C. mesoleucos*, and *C. nigropunctatus*), which provides further evidence that coral reef habitat surrounding the Arabian Peninsula may have generated a number of new taxa. Moreover, *C. dialeucos*, an Omani species, shows geographical divergence with the remaining taxa in its group (Figure 3), which all went on to colonize the Red Sea and the Arabian Gulf and must have therefore encountered contrasting environments at the western and eastern margins of their range. The shallow Arabian Gulf started to fill with seawater approximately 14,000 years ago after being dry prior to that during the last glacial maximum (Lambeck, 1996), suggesting that it was seeded by successive waves of colonization from coastal Oman. The same process would have been ongoing at the western margin of the *C. dialeucos* range, except that the conditions encountered in the Red Sea would have contrasted to those in the Arabian Gulf (DiBattista, Choat, et al., 2016). So, while there is some evidence to suggest vicariance at the scale of the Arabian Peninsula (i.e., diversification of most taxa occurred in the Plio‐Pleistocene), a stronger scenario is that natural selection driven by the major differences in environment and habitat within the area probably played an important role in the formation of endemic species assemblages (e.g., Gaither et al., 2015). Thus, even though the distribution of some of the butterflyfishes considered in the present study does stop abruptly at the entrance of the Strait of Hormuz (*Chaetodon collare*, *C. pictus*, and *C. gardneri*), it does not support the argument for geographically driven allopatry. Indeed, all of these species have a different distributional response near the other end of their distribution at the Strait of Bab al Mandab, which includes stopping before the Straits or extending through the Straits into the southern Red Sea (Figure 3). The alternative is that the incumbent widespread butterflyfish may have restricted the Red Sea to Arabian Gulf endemics from expanding further via competitive exclusion.

The current environment of the Red Sea is spatially structured with major contrasts in the cool oligotrophic waters of the northern region compared to the much higher temperatures and productivity of the southern region (i.e., Farasan Islands in Saudi Arabia to the east and Dhalak Archipelago in Eritrea to the west) (Racault et al., 2015; Raitsos et al., 2013). This shift in environmental conditions is most clearly demonstrated in the differences in life history traits associated with reef fish species that occur in both areas, but is also seen in abundance estimates across these gradients (DiBattista, Roberts, et al., 2016; Roberts et al., 2016). Such putative selection gradients are most evident in corals, which show signatures of local adaptation to divergent environmental conditions (D'Angelo et al., 2015).

### 4.2 Evaluation of the “Pleistocene extirpation” hypothesis

The second hypothesis that we tested in this study was the Pleistocene extirpation hypothesis, which predicts that all Red Sea fauna were eliminated during the last glacial maxima (~18 ka) and were only re‐populated via recent colonization events (see Biton et al., 2008). The number of species diverging at early stages in the Pleistocene disputes the argument that Red Sea fauna did not survive complete closure or restriction of water flow at the Strait of Bab al Mandab (Figure 2). Although it clearly does not coincide with a single vicariance event given the variability in the splitting dates between closely related species (Figure 3; see Michonneau, 2015 for invertebrate examples) and ancestral range reconstruction favoring +*J* parameter models (i.e., founder events between non‐adjacent ocean regions; see Table 2), glaciations likely played a role in their separation. Moreover, even though almost all sister species have small areas of overlap at their range edge, which is usually associated with allopatric speciation, in our case these do not coincide with geographical boundaries (i.e., vicariant chokepoints) such as the Strait of Bab al Mandab (see Figure 3; Lambeck, 1996; DiBattista, Choat, et al., 2016). In fact, the non‐congruent age and distribution of the endemic species indicate a series of variable events, which may reflect localized patterns of habitat and environmental change as outlined in the previous Discussion section. The best example is the relatively young clade of Arabian Peninsula endemics: *C. dialeucos*, *C. nigropunctatus*, and *C. mesoleucos* (crown node age 2.0 Ma; 2.9–1.2 Ma 95% HPD). This group appears to have been influenced by boundaries presented by the Omani coastline across areas where there are known changes in the upwelling regime (McIlwain, Claereboudt, Al‐Oufi, Zaki, & Goddard, 2005; Shi, Morrison, Bohm, & Manghnani, 2000). This is in sharp contrast to the Indo‐West Pacific parrotfishes, where present day species boundaries support the notion of allopatric divergence (Choat, Klanten, Herwerden, Robertson, & Clements, 2012), and endemics appear to have diverged into one or more subsequent endemics (i.e., secondary endemism; Rotondo, Springer, Scott, & Schlanger, 1981) based on sympatrically distributed sister‐species pairs (highlighted in Choat et al., 2012). Moreover, Red Sea endemics from most other families of reef fish appear to have equal proportions of allopatrically and sympatrically distributed sister species (Hodge et al., 2014), which is not the case for the butterflyfishes.

The diversification of these butterflyfishes occurred at a time when the coral assemblages of the world's reefs underwent a major change in coral composition and growth forms. The global proportion of staghorn coral occurrences in coral assemblages persisted throughout most of the Cenozoic but increased substantially during the Pliocene and especially the Quaternary (Renema et al. 2016). Indeed, the rapidly growing branching acroporid corals offered different structural components in terms of shelter and feeding/foraging modes when compared to massive corals such as poritids that dominated Miocene reefs more than 5 Ma. Thus, the chaetodontids of the Arabian Peninsula (particularly the corallivorous species) were exposed to a much more dynamic environment than the widespread Indo‐West Pacific species (Coles, 2003) because of their close association with sensitive coral genera that proliferated in the region.

### 4.3 Evaluation of the “ecological trait” hypothesis

The third hypothesis that we test here is whether ecological traits are linked to the evolution of endemism among butterflyfishes in the Red Sea to Arabian Gulf. We found a negative, significant relationship between endemism and depth range and, to a lesser extent, phylogenetic age for these butterflyfishes (Figures 5 and 6). The relationship between a narrow versus broad depth range and endemism supports the view that endemic species tend to be more specialized to local resources than widespread species (Hawkins, Roberts, & Clark, 2000). The majority of regional endemics in this study had depth ranges that did not extend deeper than 25 m (Figure 6), despite the availability of light dependent coral habitat extending beyond that (Kahng et al., 2010). The broad range of ages represented by these shallow water specialists suggests that adaptation to shallow reefs occurred multiple times across a relatively wide time frame (i.e., 1.3–3.3 Myr). On the other hand, speciation of endemics with a preference for deep reefs seems to be a recent phenomenon, as deeper depth ranges were strongly associated with young age (<1.75 Myr; Figure 6).

### 4.4 Comments on incomplete sampling and biogeographic biases

The goal of this study was to reconstruct the evolutionary history of Red Sea to Arabian Gulf butterflyfishes. As is the case with all phylogenetic and biogeographic reconstructions, our results have to be interpreted in light of the taxa that are not sampled, both extant and extinct. Indeed, the inclusion of missing taxa has the potential to alter lineage relationships and their age estimates, whereas their geographic distribution may alter the most likely biogeographic scenarios reconstructed across the tree (see discussion in Cowman & Bellwood, 2013). Here, we were able to sample all Red Sea to Arabian Gulf butterflyfishes (save one species, *R. jayakari*), and their close relatives from the Indian Ocean and PO, across four major chaetodontid lineages (Supporting Information Figure S2). From a temporal perspective, the topology and ages estimated for the genomic scale UCE data overlap with previous studies (Supporting Information Figures S2 and S3). Moreover, our sampling of eight species that have not previously been included in phylogenetic studies of the Chaetodontidae family means that for 13 out of the 17 Arabian Peninsular species, we are confident that we have sampled their direct sister lineage. Two of the outstanding three species (*C. melannotus*,* C. trifascialis*) are wide‐ranging Indo‐West Pacific taxa that are reconstructed to have dispersed to the Arabian Peninsula (Figure 4). The most likely sister species of *C. melannotus* is *C. ocellicaudus* (Kuiter, 2002; also see Supporting Information Figure S2), a west Pacific species not sampled in our dataset. In the case of *C. trifascialis*, it is placed as the sister lineage for a subclade of CH3 containing 10 species distributed across the Indian Ocean and PO, of which we sampled four species (Supporting Information Figure S2; Cowman & Bellwood, 2011). The final outstanding species, *C. leucopleura*, is placed as a sister species to *C. gardineri*. Both species have not previously been sampled in phylogenetic studies, but are recognized to be closely related to a third species, *Chaetodon selene* (widespread in the west Pacific, Kuiter, 2002), which was not sampled in our UCE dataset. In each of these three cases, and more broadly across the family, the inclusion of unsampled species would increase the influence of the Indian Ocean and PO in the ancestral estimation of biogeographic ranges. As such, it would act to strengthen our conclusion that even though the Red Sea and adjacent gulfs and seas have been important for the generation of endemic species, they have had little contribution to the wider Indo‐West Pacific diversity of butterflyfishes.

## 5 CONCLUSION

It appears that the unique environmental conditions in the coastal waters of the Arabian Peninsula may have contributed to the formation of endemic butterflyfishes; however, there is a lack of evidence for endemics contributing significant species richness to adjacent seas (i.e., evolutionary incubator hypothesis). Moreover, even with catastrophic environmental instability experienced by the Red Sea and coastal environments of the Arabian Peninsula due to sea level changes associated with glacial cycles (Ludt & Rocha, 2015), there is no evidence for a massive extirpation of butterflyfish fauna in the region (i.e., Pleistocene extirpation hypothesis; also see DiBattista, Choat, et al., 2016). The broad range of phylogenetic ages among endemic, shallow water butterflyfishes supports the view that species may have survived in isolated refugia within the Red Sea (DiBattista, Choat, et al., 2016). None of the dispersal‐related traits were associated with endemism, suggesting that factors other than those related to species intrinsic dispersal potential may be limiting dispersal into the greater Indian Ocean (e.g., coastline geography, oceanographic barriers).

## AUTHORS’ CONTRIBUTIONS

J.D.D., M.E.A., L.A.R., J.H.C., and M.L.B. designed the study; J.D.D., L.S., J.P.A.H., T.H.S., L.A.R., and M.L.B. collected samples; J.D.D. and L.S. generated the UCE libraries; J.D.D., M.E.A., J.C., O.J.L., and P.F.C. analyzed and interpreted data; M.F. calibrated tree reconstructions; J.D.D. wrote the manuscript with input from all co‐authors.

## DATA ACCESSIBILITY

Data associated with this manuscript are available under NCBI BioProject PRJNA484421, available at https://www.ncbi.nlm.nih.gov/bioproject/PRJNA484421.

## References

[ece34566-bib-0001] Aberer, A. J. , Kobert, K. , & Stamatakis, A. (2014). ExaBayes: Massively parallel Bayesian tree inference for the whole‐genome era. Molecular Biology and Evolution, 31, 2553–2556. 10.1093/molbev/msu236 25135941 PMC4166930

[ece34566-bib-0002] Alfaro, M. E. , Faircloth, B. C. , Harrington, R. C. , Sorenson, L. , Friedman, M. , Thacker, C. E. , … Near, T. J. (2018). Explosive diversification of marine fishes at the Cretaceous‐Palaeogene boundary. Nature Ecology & Evolution, 2, 688–696. 10.1038/s41559-018-0494-6 29531346

[ece34566-bib-0003] Allen, G. R. , Steene, R. , & Allen, M. (1998). A guide to angelfishes and butterflyfishes (250 pp). Sydney, NSW: Odyssey Publishing.

[ece34566-bib-0004] Bailey, G. (2010). The Red Sea, coastal landscapes, and hominin dispersals In The evolution of human populations in Arabia (pp. 15–37). Dordrecht, the Netherlands: Springer.

[ece34566-bib-0005] Bejerano, G. , Haussler, D. , & Blanchette, M. (2004). Into the heart of darkness: Large‐scale clustering of human non‐coding DNA. Bioinformatics, 20, i40–i48. 10.1093/bioinformatics/bth946 15262779

[ece34566-bib-0006] Bellwood, D. R. , Klanten, S. , Cowman, P. F. , Pratchett, M. S. , Konow, N. , & van Herwerden, L. (2010). Evolutionary history of the butterflyfishes (f: Chaetodontidae) and the rise of coral feeding fishes. Journal of Evolutionary Biology, 23, 335–349. 10.1111/j.1420-9101.2009.01904.x 20487131

[ece34566-bib-0007] Bianchi, C. N. , Morri, C. , Chiantore, M. , Montefalcone, M. , Parravicini, V. , & Rovere, A. (2012). Mediterranean Sea biodiversity between the legacy from the past and a future of change In Stambler N. (Ed.), Life in the Mediterranean Sea: A look at habitat changes (pp. 1–55). New York, NY: Nova Science Publishers.

[ece34566-bib-0008] Biton, E. , Gildor, H. , & Peltier, W. R. (2008). Red Sea during the Last Glacial Maximum: Implications for sea level reconstruction. Paleoceanography, 23, PA1214.

[ece34566-bib-0009] Bolger, A. M. , Lohse, M. , & Usadel, B. (2014). Trimmomatic: A flexible trimmer for Illumina Sequence Data. Bioinformatics, 30, 2114–2120. 10.1093/bioinformatics/btu170 24695404 PMC4103590

[ece34566-bib-0010] Bowen, B. W. , Rocha, L. A. , Toonen, R. J. , & Karl, S. A. (2013). The origins of tropical marine biodiversity. Trends in Ecology and Evolution, 28, 359–366. 10.1016/j.tree.2013.01.018 23453048

[ece34566-bib-0011] Castresana, J. (2000). Selection of conserved blocks from multiple alignments for their use in phylogenetic analysis. Molecular Biology and Evolution, 17, 540–552. 10.1093/oxfordjournals.molbev.a026334 10742046

[ece34566-bib-0012] Choat, J. H. , Klanten, O. S. , van Herwerden, L. , Robertson, D. R. , & Clements, K. D. (2012). Patterns and processes in the evolutionary history of parrotfishes (Family Labridae). Biological Journal of the Linnean Society, 107, 529–557. 10.1111/j.1095-8312.2012.01959.x

[ece34566-bib-0013] Cole, A. J. , & Pratchett, M. S. (2014). Diversity in diet and feeding behaviour of butterflyfishes: Reliance on reef corals versus reef habitats In Pratchett M. S., Berumen M. L., & Kapoor B. G. (Eds.), Biology of butterflyfishes (pp. 107–139). Boca Raton, FL: CRC Press.

[ece34566-bib-0014] Coles, S. L. (2003). Coral species diversity and environmental factors in the Arabian Gulf and the Gulf of Oman: A comparison to the Indo‐Pacific region. Atoll Research Bulletin, 507, 1–19.

[ece34566-bib-0015] Cowman, P. F. (2014). Historical factors that have shaped the evolution of tropical reef fishes: A review of phylogenies, biogeography, and remaining questions. Frontiers in Genetics, 5, 1–15. 25431581 10.3389/fgene.2014.00394 PMC4230204

[ece34566-bib-0016] Cowman, P. F. , & Bellwood, D. R. (2011). Coral reefs as drivers of cladogenesis: Expanding coral reefs, cryptic extinction events, and the development of biodiversity hotspots. Journal of Evolutionary Biology, 24, 2543–2562. 21985176 10.1111/j.1420-9101.2011.02391.x

[ece34566-bib-0017] Cowman, P. F. , & Bellwood, D. R. (2013). The historical biogeography of coral reef fishes: Global patterns of origination and dispersal. Journal of Biogeography, 40, 209–224.

[ece34566-bib-0018] Cowman, P. F. , Parravicini, V. , Kulbicki, M. , & Floeter, S. R. (2017). The biogeography of tropical reef fishes: Endemism and provinciality through time. Biological Reviews, 92, 2112–2130. 10.1111/brv.12323 28231621

[ece34566-bib-0019] D'Angelo, C. , Hume, B. C. C. , Burt, J. , Smith, E. G. , Achterberg, E. P. , & Wiedenmann, J. (2015). Local adaptation constrains the distribution potential of heat‐tolerant *Symbiodinium* from the Persian/Arabian Gulf. The ISME Journal, 9, 2551–2560. 10.1038/ismej.2015.80 25989370 PMC4817622

[ece34566-bib-0020] De’ath, G. , & Fabricius, K. E. (2000). Classification and regression trees: A powerful yet simple technique for ecological data analysis. Ecology, 81, 3178–3192. 10.1890/0012-9658(2000)081[3178:CARTAP]2.0.CO;2

[ece34566-bib-0021] Derti, A. , Roth, F. P. , Church, G. M. , & Wu, C. T. (2006). Mammalian ultraconserved elements are strongly depleted among segmental duplications and copy number variants. Nature Genetics, 38, 1216–1220. 10.1038/ng1888 16998490

[ece34566-bib-0022] DiBattista, J. D. , Berumen, M. L. , Gaither, M. R. , Rocha, L. A. , Eble, J. A. , Choat, J. H. , … Bowen, B. W. (2013). After continents divide: Comparative phylogeography of reef fishes from the Red Sea and Indian Ocean. Journal of Biogeography, 40, 1170–1181. 10.1111/jbi.12068

[ece34566-bib-0023] DiBattista, J. D. , Choat, J. H. , Gaither, M. R. , Hobbs, J. P. , Lozano‐Cortés, D. F. , Myers, R. F. , … Berumen, M. L. (2016). On the origin of endemic species in the Red Sea. Journal of Biogeography, 43, 13–30.

[ece34566-bib-0024] DiBattista, J. D. , Gaither, M. R. , Hobbs, J. P. A. , Saenz-Agudelo, P. , Piatek, M. J. , Bowen, B. W. , … Sinclair-Taylor, T. H. (2017). Comparative phylogeography of reef fishes from the Gulf of Aden to the Arabian Sea reveals two cryptic lineages. Coral Reefs, 36, 625–638.

[ece34566-bib-0025] DiBattista, J. D. , Roberts, M. , Bouwmeester, J. , Bowen, B. W. , Coker, D. F. , Lozano‐Cortés, D. F. , … Berumen, M. L. (2016). A review of contemporary patterns of endemism for shallow water reef fauna in the Red Sea. Journal of Biogeography, 43, 423–439.

[ece34566-bib-0026] DiBattista, J. D. , Wilcox, C. , Craig, M. T. , Rocha, L. A. , & Bowen, B. W. (2010). Phylogeography of the Pacific Blueline Surgeonfish, *Acanthurus nigroris*, reveals high genetic connectivity and a cryptic endemic species in the Hawaiian Archipelago. Journal of Marine Biology, 2011 10.1155/2011/839134

[ece34566-bib-0027] Dos Reis, M. , & Yang, Z. (2011). Approximate likelihood calculation on a phylogeny for Bayesian estimation of divergence times. Molecular Biology and Evolution, 28, 2161–2172. 10.1093/molbev/msr045 21310946

[ece34566-bib-0028] Eble, J. A. , Toonen, R. J. , Sorenson, L. , Basch, L. V. , Papastamatiou, Y. P. , & Bowen, B. W. (2011). Escaping paradise: Larval export from Hawaii in an Indo‐Pacific reef fish, the yellow tang (*Zebrasoma flavescens*). Marine Ecology Progress Series, 428, 245. 25505806 10.3354/meps09083 PMC4260458

[ece34566-bib-0029] Faircloth, B. C. (2013). illumiprocessor: A trimmomatic wrapper for parallel adapter and quality trimming. 10.6079/J9ILL

[ece34566-bib-0030] Faircloth, B. C. (2016). PHYLUCE is a software package for the analysis of conserved genomic loci. Bioinformatics, 32, 786–788. 10.1093/bioinformatics/btv646 26530724

[ece34566-bib-0031] Faircloth, B. C. , & Glenn, T. C. (2012). Not all sequence tags are created equal: Designing and validating sequence identification tags robust to indels. PLOS ONE, 7, e42543. 22900027 10.1371/journal.pone.0042543 PMC3416851

[ece34566-bib-0032] Faircloth, B. C. , McCormack, J. E. , Crawford, N. G. , Harvey, M. G. , Brumfield, R. T. , & Glenn, T. C. (2012). Ultraconserved elements anchor thousands of genetic markers spanning multiple evolutionary timescales. Systematic Biology, 61(5), 717–726. 22232343 10.1093/sysbio/sys004

[ece34566-bib-0033] Faircloth, B. C. , Sorenson, L. , Santini, F. , & Alfaro, M. E. (2013). A phylogenomic perspective on the radiation of ray‐finned fishes based upon targeted sequencing of ultraconserved elements (UCEs). PLOS ONE, 8, e65923 10.1371/journal.pone.0065923 23824177 PMC3688804

[ece34566-bib-0034] Fessler, J. L. , & Westneat, M. W. (2007). Molecular phylogenetics of the butterflyfishes (Chaetodontidae): Taxonomy and biogeography of a global coral reef fish family. Molecular Phylogenetics and Evolution, 45, 50–68. 17625921 10.1016/j.ympev.2007.05.018

[ece34566-bib-0035] Froese, R. , & Pauly, D. (2011). FishBase: World Wide Web electronic publication. Retrieved from www.fishbase.org

[ece34566-bib-0036] Gaither, M. R. , Bernal, M. A. , Coleman, R. R. , Bowen, B. W. , Jones, S. A. , Simison, W. B. , & Rocha, L. A. (2015). Genomic signatures of geographic isolation and natural selection in coral reef fishes. Molecular Ecology, 24, 1543–1557. 10.1111/mec.13129 25753379

[ece34566-bib-0037] Gaither, M. R. , Jones, S. A. , Kelley, C. , Newman, S. J. , Sorenson, L. , & Bowen, B. W. (2011). High connectivity in the deepwater snapper *Pristipomoides filamentosus* (Lutjanidae) across the Indo‐Pacific with isolation of the Hawaiian Archipelago. PLOS ONE, 6, e28913 10.1371/journal.pone.0028913 22216141 PMC3245230

[ece34566-bib-0038] Gaither, M. R. , Toonen, R. J. , Robertson, D. R. , Planes, S. , & Bowen, B. W. (2010). Genetic evaluation of marine biogeographical barriers: Perspectives from two widespread Indo‐Pacific snappers (*Lutjanus kasmira* and *Lutjanus fulvus*). Journal of Biogeography, 37, 133–147.

[ece34566-bib-0039] Girdler, R. W. , & Styles, P. (1974). Two‐stage Red Sea floor spreading. Nature, 247, 7–11.

[ece34566-bib-0040] Gnirke, A. , Melnikov, A. , Maguire, J. , Rogov, P. , LeProust, E. M. , Brockman, W. , … Russ, C. (2009). Solution hybrid selection with ultra-long oligonucleotides for massively parallel targeted sequencing. Nature Biotechnology, 27, 182–189. 10.1038/nbt.1523 PMC2663421 19182786

[ece34566-bib-0041] Grabherr, M. G. , Haas, B. J. , Yassour, M. , Levin, J. Z. , Thompson, D. A. , Amit, I. , … Regev, A. (2011). Full‐length transcriptome assembly from RNA‐seq data without a reference genome. Nature Biotechnology, 29, 644–652. 10.1038/nbt.1883 PMC3571712 21572440

[ece34566-bib-0042] Harrington, R. C. , Faircloth, B. C. , Eytan, R. I. , Smith, W. L. , Near, T. J. , Alfaro, M. E. , & Friedman, M. (2016). Phylogenomic analysis of carangimorph fishes reveals flatfish asymmetry arose in a blink of the evolutionary eye. BMC Evolutionary Biology, 16, 224 10.1186/s12862-016-0786-x 27769164 PMC5073739

[ece34566-bib-0043] Harris, R. S. (2007). Improved pairwise alignment of genomic DNA. PhD Thesis, Pennsylvania State University.

[ece34566-bib-0044] Hawkins, J. P. , Roberts, C. M. , & Clark, V. (2000). The threatened status of restricted‐range coral reef fish species. Animal Conservation, 3, 81–88. 10.1111/j.1469-1795.2000.tb00089.x

[ece34566-bib-0045] Ho, L. S. T. , Ane, C. , Lachlan, R. , Tarpinian, K. , Feldman, R. , & Ho, M. L. S. T. (2016). Package ‘phylolm’. Retrieved from ftp://videolan.c3sl.ufpr.br/CRAN/web/packages/phylolm/phylolm.pdf

[ece34566-bib-0046] Hobbs, J. P. A. , Jones, G. P. , Munday, P. L. , Connolly, S. R. , & Srinivasan, M. (2012). Biogeography and the structure of coral reef fish communities on isolated islands. Journal of Biogeography, 39, 130–139. 10.1111/j.1365-2699.2011.02576.x

[ece34566-bib-0047] Hodge, J. R. , Herwerden, L. , & Bellwood, D. R. (2014). Temporal evolution of coral reef fishes: Global patterns and disparity in isolated locations. Journal of Biogeography, 41, 2115–2127. 10.1111/jbi.12356

[ece34566-bib-0048] Hsu, K. C. , Chen, J. P. , & Shao, K. T. (2007). Molecular phylogeny of *Chaetodon* (Teleostei: Chaetodontidae) in the Indo‐West Pacific: Evolution in geminate species pairs and species groups. Raffles Bulletin of Zoology, 14, 77–86.

[ece34566-bib-0049] Hubert‐Ferrari, A. , King, G. , Manighetti, I. , Armijo, R. , Meyer, B. , & Tapponnier, P. (2003). Long‐term elasticity in the continental lithosphere; modelling the Aden Ridge propagation and the Anatolian extrusion process. Geophysical Journal International, 153, 111–132. 10.1046/j.1365-246X.2003.01872.x

[ece34566-bib-0050] Kahng, S. E. , Garcia‐Sais, J. R. , Spalding, H. L. , Brokovich, E. , Wagner, D. , Weil, E. , … Toonen, R. J. (2010). Community ecology of mesophotic coral reef ecosystems. Coral Reefs, 29, 255–275. 10.1007/s00338-010-0593-6

[ece34566-bib-0051] Katoh, K. , Misawa, K. , Kuma, K. , & Miyata, T. (2002). MAFFT: A novel method for rapid multiple sequence alignment based on fast Fourier transform. Nucleic Acids Research, 30, 3059–3066. 10.1093/nar/gkf436 12136088 PMC135756

[ece34566-bib-0052] Katoh, K. , & Standley, D. M. (2013). MAFFT multiple sequence alignment software version 7: Improvements in performance and usability. Molecular Biology and Evolution, 30, 772–780. 23329690 10.1093/molbev/mst010 PMC3603318

[ece34566-bib-0053] Keith, S. A. , Woolsey, E. S. , Madin, J. S. , Byrne, M. , & Baird, A. H. (2015). Differential establishment potential of species predicts a shift in coral assemblage structure across a biogeographic barrier. Ecography, 38, 1225–1234. 10.1111/ecog.01437

[ece34566-bib-0054] Kemp, J. (1998). Zoogeography of the coral reef fishes of the Socotra Archipelago. Journal of Biogeography, 25, 919–933. 10.1046/j.1365-2699.1998.00249.x

[ece34566-bib-0055] Klausewitz, W. (1989). Evolutionary history and zoogeography of the Red Sea ichthyofauna. Fauna of Saudi Arabia, 10, 310–337.

[ece34566-bib-0056] Kuiter, R. H. (2002). Butterflyfishes, bannerfishes and their relatives: A comprehensive guide to Chaetodontidae and Microcanthidae. Chorleywood, UK: TMC Publishing.

[ece34566-bib-0057] Lambeck, K. (1996). Shoreline reconstructions for the Persian Gulf since the last glacial maximum. Earth and Planetary Science Letters, 142, 43–57. 10.1016/0012-821X(96)00069-6

[ece34566-bib-0058] Lomolino, M. V. (2005). Body size evolution in insular vertebrates: Generality of the island rule. Journal of Biogeography, 32, 1683–1699. 10.1111/j.1365-2699.2005.01314.x

[ece34566-bib-0059] Ludt, W. B. , & Rocha, L. A. (2015). Shifting seas: The impacts of Pleistocene sea‐level fluctuations on the evolution of tropical marine taxa. Journal of Biogeography, 42, 25–38.

[ece34566-bib-0060] Luiz, O. J. , Allen, A. P. , Robertson, D. R. , Floeter, S. R. , Kulbicki, M. , Vigliola, L. , … Madin, J. S. (2013). Adult and larval traits as determinants of geographic range size among tropical reef fishes. Proceedings of National Academy of Sciences U.S.A., 110, 16498–16502. 10.1073/pnas.1304074110 PMC3799316 24065830

[ece34566-bib-0061] Luiz, O. J. , Madin, J. S. , Robertson, D. R. , Rocha, L. A. , Wirtz, P. , & Floeter, S. R. (2012). Ecological traits influencing range expansion across large oceanic dispersal barriers: Insights from tropical Atlantic reef fishes. Proceedings of the Royal Society B, 279, 1033–1040. 10.1098/rspb.2011.1525 21920979 PMC3259933

[ece34566-bib-0062] Malay, M. C. M. D. , & Paulay, G. (2010). Peripatric speciation drives diversification and distributional pattern of reef hermit crabs (Decapoda: Diogenidae: *Calcinus*). Evolution, 64, 634–662. 19796150 10.1111/j.1558-5646.2009.00848.x

[ece34566-bib-0063] Matzke, N. J. (2013). Probabilistic historical biogeography: New models for founder‐event speciation, imperfect detection, and fossils allow improved accuracy and model‐testing. Frontiers in Biogeography, 4, 210.

[ece34566-bib-0064] McCormack, J. E. , Harvey, M. G. , Faircloth, B. C. , Crawford, N. G. , Glenn, T. C. , & Brumfield, R. T. (2013). A phylogeny of birds based on over 1,500 loci collected by target enrichment and high‐throughput sequencing. PLOS ONE, 8, e54848 10.1371/journal.pone.0054848 23382987 PMC3558522

[ece34566-bib-0065] McGee, M. D. , Faircloth, B. C. , Borstein, S. R. , Zheng, J. , Darrin Hulsey, C. , Wainwright, P. C. , & Alfaro, M. E. (2016). Replicated divergence in cichlid radiations mirrors a major vertebrate innovation. Proceedings of the Royal Society B, 283, 20151413. 26763694 10.1098/rspb.2015.1413 PMC4721080

[ece34566-bib-0066] McIlwain, J. L. , Claereboudt, M. R. , Al‐Oufi, H. S. , Zaki, S. , & Goddard, J. S. (2005). Spatial variation in age and growth of the kingfish (*Scomberomorus commerson*) in the coastal waters of the Sultanate of Oman. Fisheries Research, 73, 283–298. 10.1016/j.fishres.2004.10.020

[ece34566-bib-0067] McKinney, M. L. (1997). Extinction vulnerability and selectivity: Combining ecological and paleontological views. Annual Review of Ecology and Systematics, 28, 495–516. 10.1146/annurev.ecolsys.28.1.495

[ece34566-bib-0068] Michonneau, F. (2015). Cryptic and not‐so‐cryptic species in the complex “holothuria (Thymiosycia) imaptiens” (Forsskål, 1775) (Echinodermata: Holothuroidea: Holothuriidae). Biorxiv., 014225 10.1101/014225

[ece34566-bib-0069] Ottimofiore, E. , Albouy, C. , Leprieur, F. , Descombes, P. , Kulbicki, M. , Mouillot, D. , … Pellissier, L. (2017). Responses of coral reef fishes to past climate changes are related to life‐history traits. Ecology and Evolution, 7, 1996–2005. 10.1002/ece3.2800 28331606 PMC5355194

[ece34566-bib-0070] Pous, S. , Lazure, P. , & Carton, X. (2015). A model of the general circulation in the Persian Gulf and in the Strait of Hormuz: Intraseasonal to interannual variability. Continental Shelf Research, 94, 55–70. 10.1016/j.csr.2014.12.008

[ece34566-bib-0071] Pratchett, M. S. (2014). Feeding preferences and dietary specialisation among obligate coral‐feeding butterflyfishes In Pratchett M. S., Berumen M. L., & Kapoor B. G. (Eds.), Biology of butterflyfishes (pp. 140–179). Boca Raton, FL: CRC Press.

[ece34566-bib-0072] Racault, M. F. , Raitsos, D. E. , Berumen, M. L. , Brewin, R. J. , Platt, T. , Sathyendranath, S. , & Hoteit, I. (2015). Phytoplankton phenology indices in coral reef ecosystems: Application to ocean‐color observations in the Red Sea. Remote Sensing of Environment, 160, 222–234. 10.1016/j.rse.2015.01.019

[ece34566-bib-0073] Raitsos, D. E. , Pradhan, Y. , Brewin, R. J. W. , Stenchikov, G. , & Hoteit, I. (2013). Remote sensing the phytoplankton seasonal succession of the Red Sea. PLOS ONE, 8, e64909 10.1371/journal.pone.0064909 23755161 PMC3674012

[ece34566-bib-0074] Rambaut, A. , Suchard, M. A. , Xie, D. , & Drummond, A. J. (2014). Tracer v1.6. Retrieved from https://beast.bio.ed.ac.uk/Tracer

[ece34566-bib-0075] Reneker , J. , Lyons , E. , Conant , G. C. , … D. (2012). Long identical multispecies elements in plant and animal genomes. Proceedings of the National Academy of Sciences U.S.A., 109, E1183–E1191. 10.1073/pnas.1121356109 PMC3358895 22496592

[ece34566-bib-0076] Renema, W. , Pandolfi, J. M. , Kiessling, W. , Bosellini, F. R. , Klaus, J. S. , Korpanty, C. , … Johnson, K. G. (2016). Are coral reefs victims of their own past success?. Science Advances, 2, e1500850. 27152330 10.1126/sciadv.1500850 PMC4846430

[ece34566-bib-0077] Roberts, M. B. , Jones, G. P. , McCormick, M. I. , Munday, P. L. , Neale, S. , Thorrold, S. , … Berumen, M. L. (2016). Homogeneity of coral reef communities across 8 degrees of latitude in the Saudi Arabian Red Sea. Marine Pollution Bulletin, 105, 558–565. 10.1016/j.marpolbul.2015.11.024 26608504

[ece34566-bib-0078] Rohling, E. J. , Grant, K. , Bolshaw, M. , Roberts, A. P. , Siddall, M. , Hemleben, C. , & Kucera, M. (2009). Antarctic temperature and global sea level closely coupled over the past five glacial cycles. Nature Geoscience, 2, 500–504. 10.1038/ngeo557

[ece34566-bib-0079] Rotondo, G. M. , Springer, V. G. , Scott, G. A. , & Schlanger, S. O. (1981). Plate movement and island integration—a possible mechanism in the formation of endemic biotas, with special reference to the Hawaiian Islands. Systematic Biology, 30, 12–21.

[ece34566-bib-0080] Shi, W. , Morrison, J. M. , Bohm, E. , & Manghnani, V. (2000). The Oman upwelling zone during 1993, 1994 and 1995. Deep‐Sea Research II, 47, 1227–1247. 10.1016/S0967-0645(99)00142-3

[ece34566-bib-0081] Siddall, M. , Rohling, E. J. , Almogi‐Labin, A. , Hemleben, C. , Meischner, D. , Schmelzer, I. , & Smeed, D. A. (2003). Sea‐level fluctuations during the last glacial cycle. Nature, 423, 853–858. 10.1038/nature01690 12815427

[ece34566-bib-0082] Siepel, A. , Bejerano, G. , Pedersen, J. S. , Hinrichs, A. S. , Hou, M. , Rosenbloom, K. , … Haussler, D. (2005). Evolutionarily conserved elements in vertebrate, insect, worm, and yeast genomes. Genome Research, 15, 1034–1050. 10.1101/gr.3715005 16024819 PMC1182216

[ece34566-bib-0083] Simons, C. , Pheasant, M. , Makunin, I. V. , & Mattick, J. S. (2006). Transposon‐free regions in mammalian genomes. Genome Research, 16, 164–172. 16365385 10.1101/gr.4624306 PMC1361711

[ece34566-bib-0084] Skillings, D. J. , Bird, C. E. , & Toonen, R. J. (2010). Gateways to Hawai‘i: Genetic population structure of the tropical sea cucumber *Holothuria atra* . Journal of Marine Biology, 2011 10.1155/2011/783030

[ece34566-bib-0085] Smeed, D. (1997). Seasonal variation of the flow in the strait of Bah al Mandab. Acta Oceanologica, 20, 773–781.

[ece34566-bib-0086] Smith, B. T. , McCormack, J. E. , Cuervo, A. M. , Hickerson, M. J. , Aleixo, A. , Cadena, C. D. , … Brumfield, R. T. (2014). The drivers of tropical speciation. Nature, 515, 406–409. 10.1038/nature13687 25209666

[ece34566-bib-0087] Sun, K. , Meiklejohn, K. A. , Faircloth, B. C. , Glenn, T. C. , Braun, E. L. , & Kimball, R. T. (2014). The evolution of peafowl and other taxa with ocelli (eyespots): A phylogenomic approach. Proceedings of the Royal Society B, 281, 20140823 10.1098/rspb.2014.0823 25030982 PMC4123699

[ece34566-bib-0088] Talavera, G. , & Castresana, J. (2007). Improvement of phylogenies after removing divergent and ambiguously aligned blocks from protein sequence alignments. Systematic Biology, 56, 564–577. 10.1080/10635150701472164 17654362

[ece34566-bib-0089] Therneau, T. , Atkinson, B. , & Ripley, B. (2015). ‘rpart’. R package version 4.1‐10.

[ece34566-bib-0090] Waldrop, E. , Hobbs, J. P. A. , Randall, J. E. , DiBattista, J. D. , Rocha, L. A. , Kosaki, R. K. , … Bowen, B. W. (2016). Phylogeography, population structure and evolution of coral-eating butterflyfishes (Family Chaetodontidae, genus Chaetodon, subgenus Corallochaetodon). Journal of Biogeography, 43, 1116–1129.

[ece34566-bib-0091] Whittaker, R. J. , & Fernández‐Palacios, J. M. (2007). Island biogeography: Ecology, evolution, and conservation. Oxford, UK: Oxford University Press.

[ece34566-bib-0092] Yabuta, S. , & Berumen, M. L. (2013). Social structures and spawning behaviour of *Chaetodon* butterflyfishes In Pratchett M. S., Berumen M. L., & Kapoor B. G. (Eds.), Biology of Butterflyfishes (pp. 200–225). Boca Raton, FL: CRC Press.

