# Supplementary figures and images for: Ice ages and butterflyfishes: Phylogenomics elucidates the ecological and evolutionary history of reef fishes in an endemism hotspot
Source: Ecol Evol. 2018 Oct 23;8(22):10989–1008. doi: 10.1002/ece3.4566 (PMC6262737; doi:10.1002/ece3.4566)

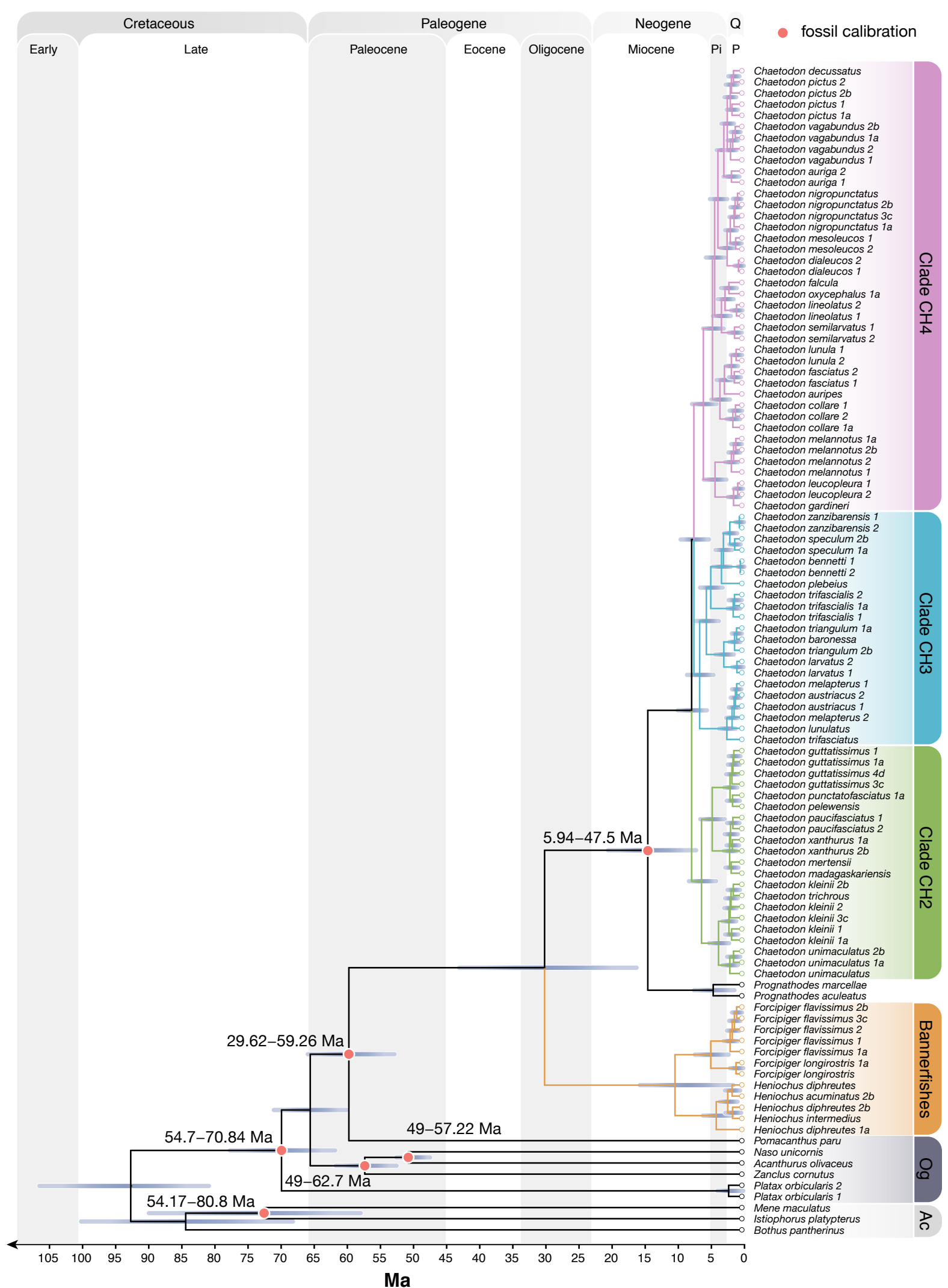

Supplement: Supplementary file 1 [file ECE3-8-10989-s001.pdf]

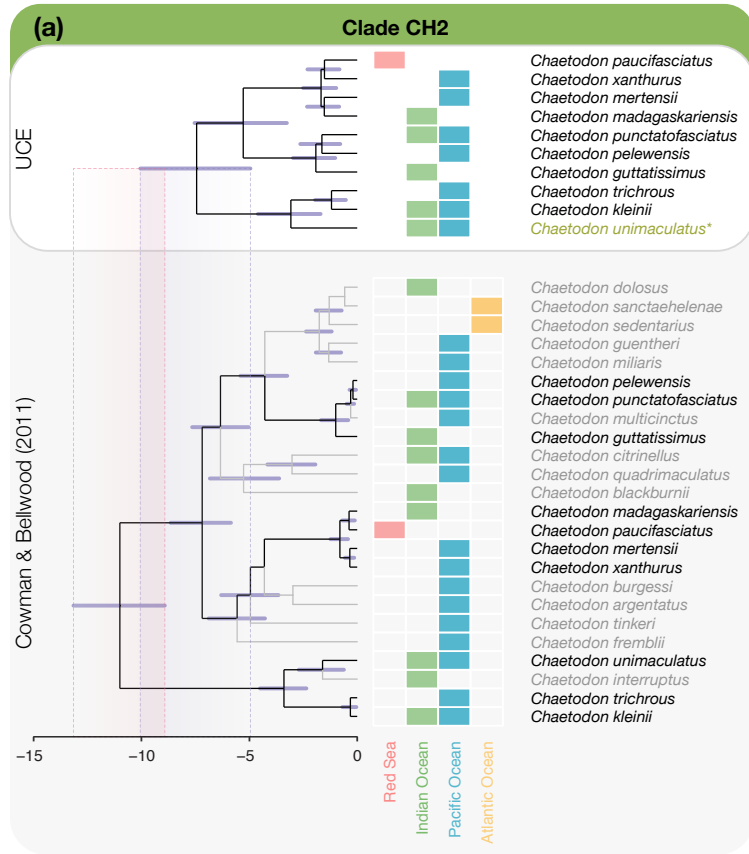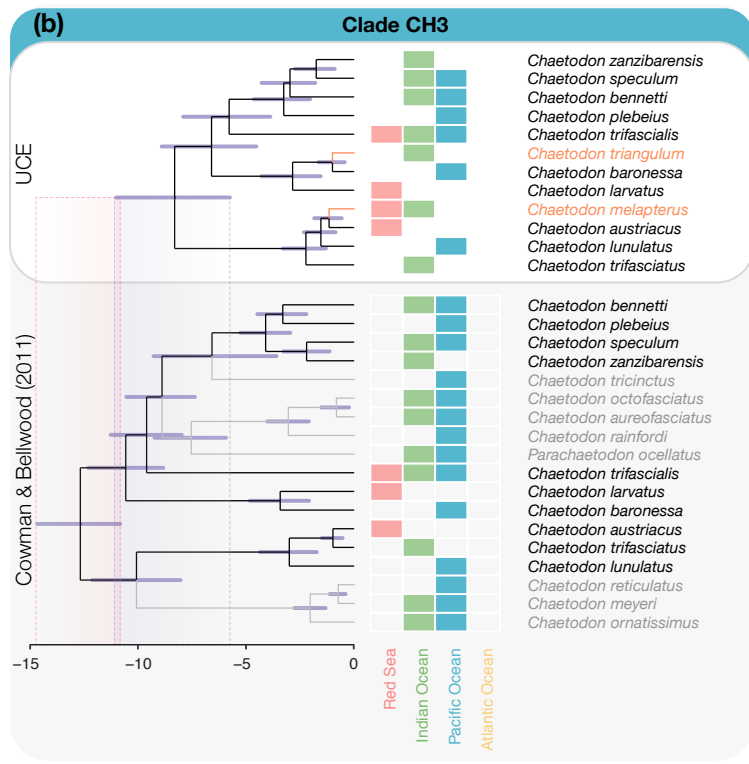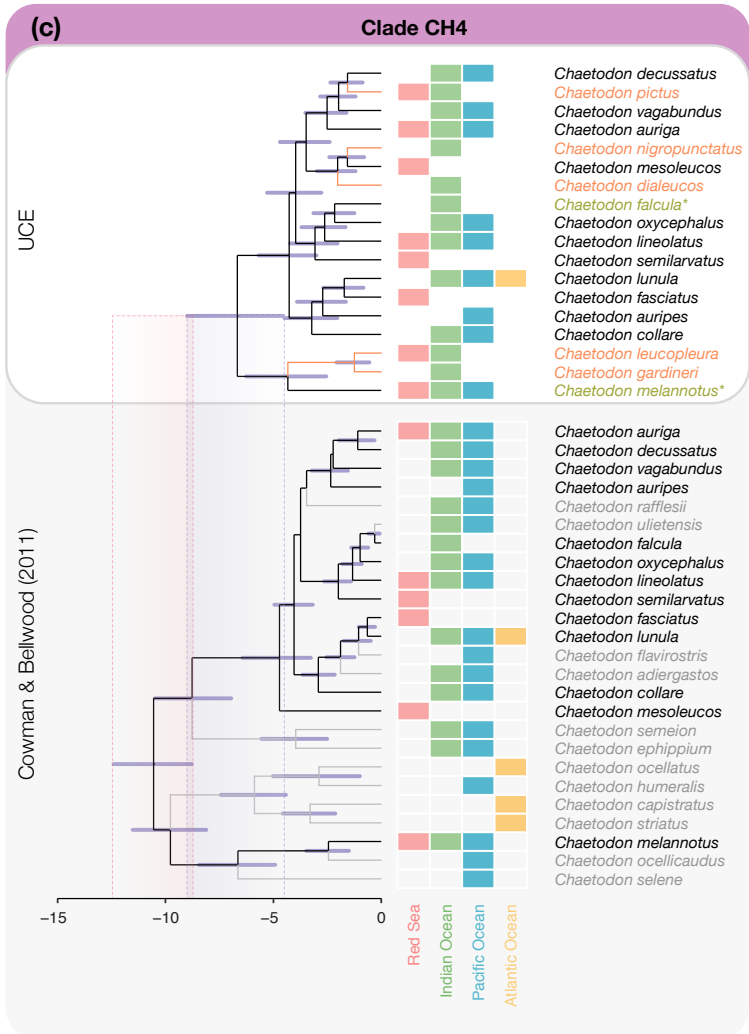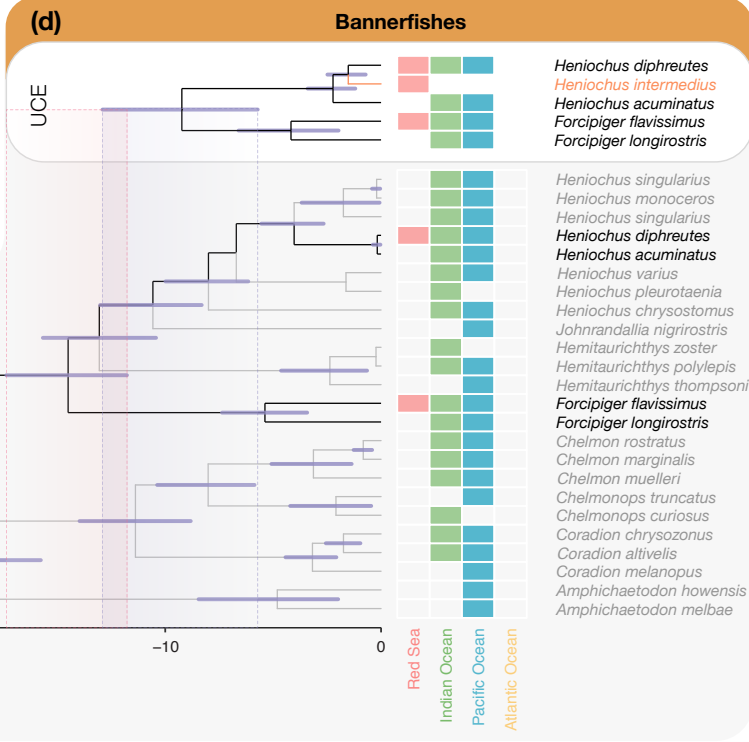

Supplement: Supplementary file 2 [file ECE3-8-10989-s002.pdf]

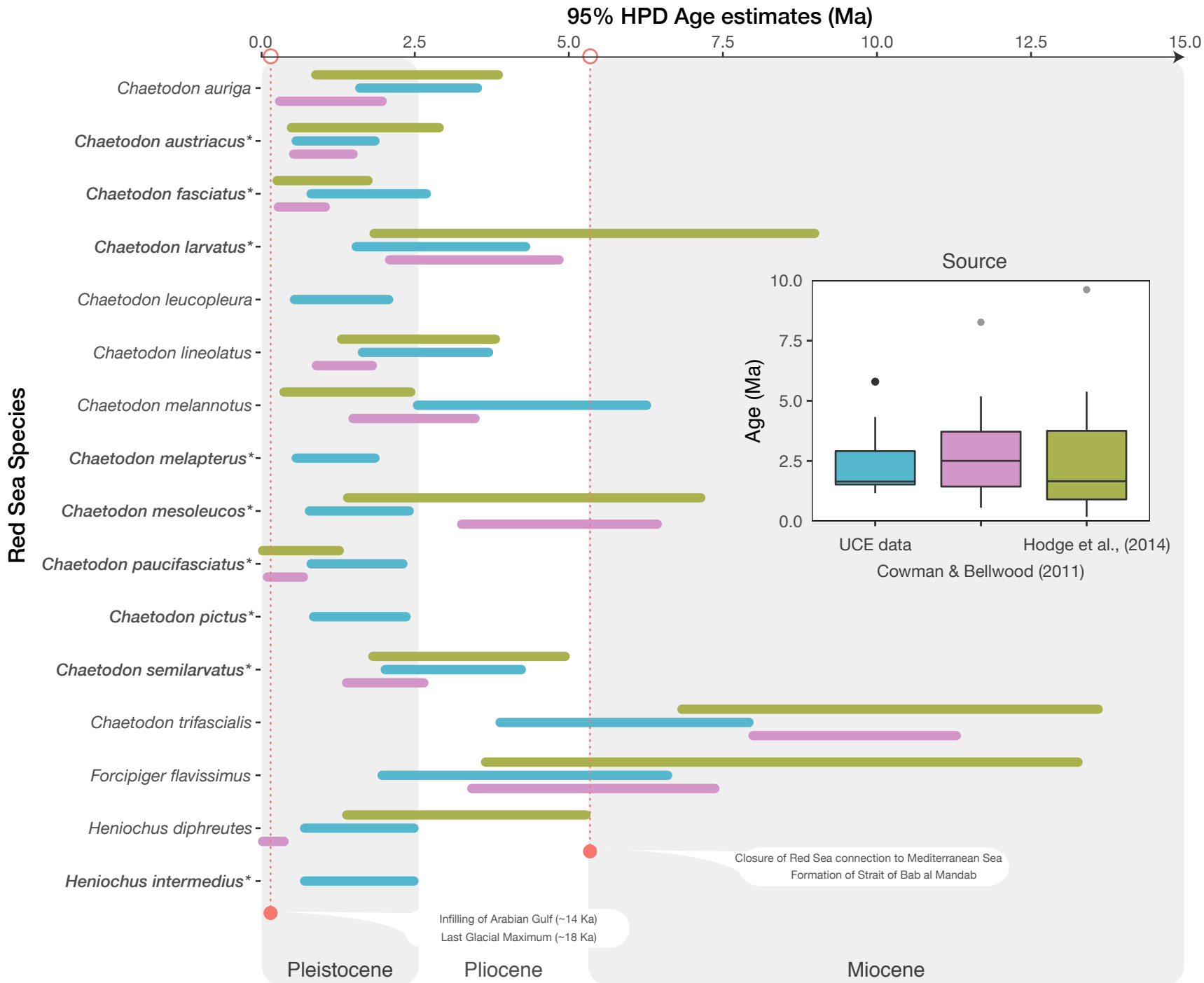

Supplement: Supplementary file 3 [file ECE3-8-10989-s003.pdf]
