# Supplementary material for: Ice ages and butterflyfishes: Phylogenomics elucidates the ecological and evolutionary history of reef fishes in an endemism hotspot
Source: Ecol Evol. 2018 Oct 23;8(22):10989–1008. doi: 10.1002/ece3.4566 (PMC6262737; doi:10.1002/ece3.4566)
Supplement: Supplementary file 4 [file ECE3-8-10989-s004.docx]

**Table S1.** Individual-level information (where available) on species, sample ID, sampling locality (including latitude and longitude), as well as taxonomic remarks.

| Species | UCE_ID | Tissue ID | Date | Locality | Latitude | Longitude | Taxonomic remarks |
| --- | --- | --- | --- | --- | --- | --- | --- |
| *Acanthurus olivaceus* | Acanthurus_olivaceus | MEA878 | December 16, 2010 | n/a | n/a | n/a | Outgroup, purchased from Quality Marine |
| *Bothus pantherinus* | Bothus_pantherinus | n/a | n/a | n/a | n/a | n/a | Outgroup, data obtained from Harrington et al. (2016) |
| *Chaetodon auriga* | Chaetodon_auriga1 | RS5441 | September 30, 2013 | Gulf of Aqaba, Saudi Arabia | 28.40387 | 34.74067 |  |
| *Chaetodon auriga* | Chaetodon_auriga2 | RS5915 | October 11, 2013 | Socotra, Yemen | 12.6717 | 54.19608 |  |
| *Chaetodon auripes* | Chaetodon_auripes | 19 | June, 2013 | Okinawa, Japan | n/a | n/a |  |
| *Chaetodon austriacus* | Chaetodon_austriacus1 | RS5334 | September 29, 2013 | Gulf of Aqaba, Saudi Arabia | 28.40387 | 34.74067 |  |
| *Chaetodon austriacus* | Chaetodon_austriacus2 | RS3553 | March 5, 2013 | Farasan Banks, Saudi Arabia | 18.658617 | 40.826967 | Vouchered at the California Academy of Sciences (CAS236290) |
| *Chaetodon baronessa* | Chaetodon_baronessa | 215088 | June 29, 2013 | Fiji | -16.810139 | 179.47575 | Vouchered at the American Museum of Natural History (I-260886) |
| *Chaetodon bennetti* | Chaetodon_bennetti1 | 3A | 2005 | Marshall Islands | n/a | n/a |  |
| *Chaetodon bennetti* | Chaetodon_bennetti2 | RS5997 | October 12, 2013 | Socotra, Yemen | 12.67033 | 54.17802 |  |
| *Chaetodon collare* | Chaetodon_collare1a | RS7171 | May 12, 2014 | Faafu Atoll, Maldives | 3.075361 | 72.965231 | Vouchered at the California Academy of Sciences (CAS237258) |
| *Chaetodon collare* | Chaetodon_collare1 | RS4767 | March 28, 2013 | Muscat, Oman | 23.68362 | 58.5019 |  |
| *Chaetodon collare* | Chaetodon_collare2 | RS6035 | October 12, 2015 | Socotra, Yemen | 12.66757 | 54.17927 |  |
| *Chaetodon decussatus* | Chaetodon_decussatus | CDE100 | 2013 | South Asia | n/a | n/a | Purchased at aquarium dealer in Los Angeles, USA |
| *Chaetodon dialeucos* | Chaetodon_dialeucos1 | RS4179 | March 19, 2013 | Mirbat, Oman | 16.945417 | 54.806611 |  |
| *Chaetodon dialeucos* | Chaetodon_dialeucos2 | RS4509 | March 26, 2013 | Masirah Island, Oman | 20.188806 | 58.626583 |  |
| *Chaetodon falcula* | Chaetodon_falcula | Cha_131 | February 26, 2013 | Salomon Atoll, British Indian Ocean Territory | n/a | n/a |  |
| *Chaetodon fasciatus* | Chaetodon_fasciatus1 | RS5275 | September 29, 2013 | Gulf of Aqaba, Saudi Arabia | 28.40387 | 34.74067 |  |
| *Chaetodon fasciatus* | Chaetodon_fasciatus2 | RS1586 | September 27, 2012 | Maskali, Djibouti | 11.699183 | 43.1432 | Vouchered at the California Academy of Sciences (CAS235008) |
| *Chaetodon cf. gardineri* | Chaetodon_gardineri | RS6242 | October 15, 2013 | Socotra, Yemen | 12.619317 | 54.3551 | Vouchered at the California Academy of Sciences (CAS236942); Putative hybrid between *C. gardineri* x *C. leucopleura* |
| *Chaetodon guttatissimus* | Chaetodon_guttatissimus1a | Cha_006 | February 24, 2013 | Diego Garcia, British Indian Ocean Territory | n/a | n/a | Vouchered at the California Academy of Sciences (CAS235915) |
| *Chaetodon guttatissimus* | Chaetodon_guttatissimus2b | RS6101 | October 13, 2013 | Socotra, Yemen | 12.66765 | 54.17927 | Vouchered at the California Academy of Sciences (CAS236913) |
| *Chaetodon guttatissimus* | Chaetodon_guttatissimus3c | RS7049 | May 11, 2014 | Faafu Atoll, Maldives | 3.075361 | 72.965231 |  |
| *Chaetodon guttatissimus* | Chaetodon_guttatissimus4d | Cgut55 | n/a | Christmas Island, Australia | n/a | n/a |  |
| *Chaetodon guttatissimus* | Chaetodon_guttatissimus1 | Cha_040 | February 25, 2013 | Salomon Atoll, British Indian Ocean Territory | n/a | n/a |  |
| *Chaetodon interruptus* | Chaetodon_interruptus1a | M1456 | November 27, 2003 | Île Rodrigues, Mauritius | n/a | n/a |  |
| *Chaetodon kleinii* | Chaetodon_kleinii1a | RS6944 | May 9, 2014 | Faafu Atoll, Maldives | 3.075361 | 72.965231 | Vouchered at the California Academy of Sciences (CAS237260) |
| *Chaetodon kleinii* | Chaetodon_kleinii2b | RS6997 | May 10, 2014 | Faafu Atoll, Maldives | 3.075361 | 72.965231 | Vouchered at the California Academy of Sciences (CAS237261) |
| *Chaetodon kleinii* | Chaetodon_kleinii3c | LAR2147 | May 9, 2014 | Sombrero Island, Philippines | n/a | n/a |  |
| *Chaetodon kleinii* | Chaetodon_kleinii1 | Cha_046 | February 25, 2013 | Salomon Atoll, British Indian Ocean Territory | n/a | n/a | Vouchered at the California Academy of Sciences (CAS235932) |
| *Chaetodon kleinii* | Chaetodon_kleinii2 | RS5998 | October 12, 2013 | Socotra, Yemen | 12.67033 | 54.17802 | Missing white bar and deep orange colour |
| *Chaetodon larvatus* | Chaetodon_larvatus1 | RS1373 | March 23, 2012 | Yanbu, Saudi Arabia | 24.2 | 37.91667 |  |
| *Chaetodon larvatus* | Chaetodon_larvatus2 | RS1555 | September 27, 2012 | Maskali, Djibouti | 11.69918 | 43.14322 |  |
| *Chaetodon leucopleura* | Chaetodon_leucopleura1 | RS4197 | March 19, 2013 | Mirbat, Oman | 16.95025 | 54.818528 |  |
| *Chaetodon leucopleura* | Chaetodon_leucopleura2 | RS6251 | October 16, 2013 | Socotra, Yemen | 12.6048 | 54.35087 |  |
| *Chaetodon lineolatus* | Chaetodon_lineolatus1a | RS7266 | May 13, 2014 | Faafu Atoll, Maldives | 3.075361 | 72.965231 | Vouchered at the California Academy of Sciences (CAS237262) |
| *Chaetodon lineolatus* | Chaetodon_lineolatus1 | RS3822 | May 8, 2013 | Farasan Banks, Saudi Arabia | 18.07308 | 40.8859 |  |
| *Chaetodon lineolatus* | Chaetodon_lineolatus2 | RS6277 | October 16, 2013 | Socotra, Yemen | 12.6717 | 54.19608 |  |
| *Chaetodon lunula* | Chaetodon_lunula1a | RS7283 | May 14, 2014 | Faafu Atoll, Maldives | 3.075361 | 72.965231 |  |
| *Chaetodon lunula* | Chaetodon_lunula1 | RS5960 | October 12, 2013 | Socotra, Yemen | 12.6717 | 54.19608 |  |
| *Chaetodon lunula* | Chaetodon_lunula2 | Cln1 | January 6, 2006 | Big Island, USA | n/a | n/a |  |
| *Chaetodon lunulatus* | Chaetodon_lunulatus | Clu394 | August 1, 2008 | Nukunonu, Tokelau Islands | n/a | n/a |  |
| *Chaetodon madagaskariensis* | Chaetodon_madagaskariensis | Cma11 | 2011 | Madagascar | n/a | n/a |  |
| *Chaetodon melannotus* | Chaetodon_melannotus1a | PI_049 | December 4, 2013 | Maricaban Island, Philippines | n/a | n/a |  |
| *Chaetodon melannotus* | Chaetodon_melannotus2b | PI_307 | December 6, 2013 | Sombrero Island, Philippines | n/a | n/a |  |
| *Chaetodon melannotus* | Chaetodon_melannotus1 | RS5467 | September 30, 2013 | Gulf of Aqaba, Saudi Arabia | 28.40387 | 34.74067 |  |
| *Chaetodon melannotus* | Chaetodon_melannotus2 | RS6189 | October 15, 2013 | Socotra, Yemen | 12.6048 | 54.35087 |  |
| *Chaetodon melapterus* | Chaetodon_melapterus1 | RS1541 | September 27, 2012 | Maskali, Djibouti | 11.699183 | 43.1432 | Vouchered at the California Academy of Sciences (CAS235003) |
| *Chaetodon melapterus* | Chaetodon_melapterus2 | RS4650 | March 27, 2013 | Muscat, Oman | 23.526972 | 58.739833 |  |
| *Chaetodon mertensii* | Chaetodon_mertensii | MCE_0385 | June, 2012 | Raratonga, Cook Islands | n/a | n/a |  |
| *Chaetodon mesoleucos* | Chaetodon_mesoleucos1 | RS1451 | March 26, 2012 | Yanbu, Saudi Arabia | 23.87428 | 38.07495 |  |
| *Chaetodon mesoleucos* | Chaetodon_mesoleucos2 | RS1570 | September 27, 2012 | Maskali, Djibouti | 11.69918 | 43.14322 |  |
| *Chaetodon nigropunctatus* | Chaetodon_nigropunctatus | RS4484 | March 26, 2013 | Masirah Island, Oman | 20.188806 | 58.626583 |  |
| *Chaetodon nigropunctatus* | Chaetodon_nigropunctatus1a | RS4568 | March 26, 2013 | Masirah Island, Oman | 20.207361 | 58.630389 |  |
| *Chaetodon nigropunctatus* | Chaetodon_nigropunctatus2b | RS4569 | March 26, 2013 | Masirah Island, Oman | 20.207361 | 58.630389 |  |
| *Chaetodon nigropunctatus* | Chaetodon_nigropunctatus3c | RS4570 | March 26, 2013 | Masirah Island, Oman | 20.207361 | 58.630389 |  |
| *Chaetodon oxycephalus* | Chaetodon_oxycephalus1a | RS6941 | May 9, 2014 | Faafu Atoll, Maldives | 3.075361 | 72.965231 | Vouchered at the California Academy of Sciences (CAS237267) |
| *Chaetodon paucifasciatus* | Chaetodon_paucifasciatus1 | RS5309 | September 29, 2013 | Gulf of Aqaba, Saudi Arabia | 28.40387 | 34.74067 |  |
| *Chaetodon paucifasciatus* | Chaetodon_paucifasciatus2 | RS3591 | March 5, 2013 | Farasan Banks, Saudi Arabia | 18.658617 | 40.826967 | Vouchered at the California Academy of Sciences (CAS236294) |
| *Chaetodon pelewensis* | Chaetodon_pelewensis | Cpel2 | June, 2012 | Raratonga, Cook Islands | n/a | n/a |  |
| *Chaetodon pictus* | Chaetodon_pictus1a | RS4121 | March 18, 2013 | Salalah, Oman | 16.912306 | 53.957194 | Vouchered at the California Academy of Sciences (CAS237548) |
| *Chaetodon pictus* | Chaetodon_pictus2b | RS5922 | October 11, 2013 | Socotra, Yemen | 12.6717 | 54.19608 |  |
| *Chaetodon pictus* | Chaetodon_pictus1 | RS1567 | September 27, 2012 | Maskali, Djibouti | 11.6991 | 43.14322 |  |
| *Chaetodon pictus* | Chaetodon_pictus2 | RS4814 | March 28, 2013 | Muscat, Oman | 23.68368 | 58.50053 |  |
| *Chaetodon plebeius* | Chaetodon_plebeius | 215086 | June 29, 2013 | Fiji | -16.810139 | 179.47575 | Vouchered at the American Museum of Natural History (I-260885) |
| *Chaetodon punctatofasciatus* | Chaetodon_punctatofasciatus1a | PI_611 | December 10, 2013 | Sombrero Island, Philippines | n/a | n/a |  |
| *Chaetodon semilarvatus* | Chaetodon_semilarvatus1 | RS5326 | September 29, 2013 | Gulf of Aqaba, Saudi Arabia | 28.40387 | 34.74067 |  |
| *Chaetodon semilarvatus* | Chaetodon_semilarvatus2 | RS1579 | September 27, 2012 | Maskali, Djibouti | 11.69918 | 43.14322 |  |
| *Chaetodon speculum* | Chaetodon_speculum1a | PI_261 | December 6, 2013 | Sombrero Island, Philippines | n/a | n/a |  |
| *Chaetodon speculum* | Chaetodon_speculum2b | PI_636 | December 10, 2013 | Sombrero Island, Philippines | n/a | n/a |  |
| *Chaetodon triangulum* | Chaetodon_triangulum1a | RS6893 | May 8, 2014 | Faafu Atoll, Maldives | 3.075361 | 72.965231 | Vouchered at the California Academy of Sciences (CAS237268) |
| *Chaetodon triangulum* | Chaetodon_triangulum2b | RS6945 | May 9, 2014 | Faafu Atoll, Maldives | 3.075361 | 72.965231 | Vouchered at the California Academy of Sciences (CAS237269) |
| *Chaetodon trichrous* | Chaetodon_trichrous | Ctrich1 | 2010 | Nuku Hiva, French Polynesia | n/a | n/a |  |
| *Chaetodon trifascialis* | Chaetodon_trifascialis1a | RS7173 | May 12, 2014 | Faafu Atoll, Maldives | 3.075361 | 72.965231 | Vouchered at the California Academy of Sciences (CAS237272) |
| *Chaetodon trifascialis* | Chaetodon_trifascialis1 | RS5354 | September 29, 2013 | Gulf of Aqaba, Saudi Arabia | 28.40387 | 34.74067 |  |
| *Chaetodon trifascialis* | Chaetodon_trifascialis2 | Cha_044 | February 25, 2013 | Salomon Atoll, British Indian Ocean Territory | n/a | n/a | Vouchered at the California Academy of Sciences (CAS235940) |
| *Chaetodon trifasciatus* | Chaetodon_trifasciatus | Cti5 | 2011 | Madagascar | n/a | n/a |  |
| *Chaetodon ulietensis* | Chaetodon_ulietensis1a | M1639 | n/a | One Tree Island, Australia | n/a | n/a | Vouchered at James Cook University (10281.3) |
| *Chaetodon unimaculatus* | Chaetodon_unimaculatus | NW13_245 | May 30, 2015 | Johnston Atoll | n/a | n/a |  |
| *Chaetodon unimaculatus* | Chaetodon_unimaculatus1a | LAR 2142 | May 9, 2014 | Sombrero Island, Philippines | n/a | n/a |  |
| *Chaetodon unimaculatus* | Chaetodon_unimaculatus2b | LAR 2143 | May 9, 2014 | Sombrero Island, Philippines | n/a | n/a |  |
| *Chaetodon vagabundus* | Chaetodon_vagabundus1a | PI_310 | December 6, 2013 | Sombrero Island, Philippines | n/a | n/a |  |
| *Chaetodon vagabundus* | Chaetodon_vagabundus2b | PI_635 | December 10, 2013 | Sombrero Island, Philippines | n/a | n/a |  |
| *Chaetodon vagabundus* | Chaetodon_vagabundus1 | Cva3 | 2011 | Madagascar | n/a | n/a |  |
| *Chaetodon vagabundus* | Chaetodon_vaganbundus2 | 215096 | July 1, 2013 | Fiji | -16.807528 | 179.468972 | Vouchered at the American Museum of Natural History (I-260889) |
| *Chaetodon xanthurus* | Chaetodon_xanthurus1a | PI_048 | December 4, 2013 | Maricaban Island, Philippines | n/a | n/a |  |
| *Chaetodon xanthurus* | Chaetodon_xanthurus2b | PI_156 | December 5, 2013 | Luzon, Philippines | n/a | n/a |  |
| *Chaetodon zanzibariensis* | Chaetodon_zanzibariensis1 | Cha_162 | February 27, 2013 | Salomon Atoll, British Indian Ocean Territory | n/a | n/a | Vouchered at the California Academy of Sciences (CAS235943) |
| *Chaetodon zanzibariensis* | Chaetodon_zanzibariensis2 | RS6027 | October 12, 2013 | Socotra, Yemen | 12.66757 | 54.17927 |  |
| *Forcipiger flavissimus* | Forcipiger_flavissimus1a | RS7041 | May 11, 2014 | Faafu Atoll, Maldives | 3.075361 | 72.965231 |  |
| *Forcipiger flavissimus* | Forcipiger_flavissimus2b | XMAS_51 | May 1, 2014 | Christmas Island, Australia | -10.45533 | 105.5692 |  |
| *Forcipiger flavissimus* | Forcipiger_flavissimus3c | XMAS_52 | May 1, 2014 | Christmas Island, Australia | -10.45533 | 105.5692 |  |
| *Forcipiger flavissimus* | Forcipiger_flavissimus1 | NW13_439 | May 30, 2013 | Johnston Atoll, USA | n/a | n/a |  |
| *Forcipiger flavissimus* | Forcipiger_flavissimus2 | RS6140 | October 14, 2013 | Socotra, Yemen | 12.617717 | 54.35475 | Vouchered at the California Academy of Sciences (CAS236926) |
| *Forcipiger longirostris* | Forcipiger_longirostris | FOL2 | n/a | Maui, USA | n/a | n/a |  |
| *Forcipiger longirostris* | Forcipiger_longirostris1a | XMAS_50 |  | Christmas Island, Australia | -10.46633 | 105.6067 | Black morph |
| *Heniochus acuminatus* | Heniochus_acuminatus2b | PI_123 | December 4, 2013 | Maricaban Island, Philippines | n/a | n/a |  |
| *Heniochus acuminatus* | Heniochus_acuminatus | RS6013 | October 12, 2013 | Socotra, Yemen | 12.670333 | 54.178017 | Vouchered at the California Academy of Sciences (CAS236896); 11 dorsal spines and long snout |
| *Heniochus diphreutes* | Heniochus_diphreutes1a | RS7394 | May 17, 2014 | Faafu Atoll, Maldives | 3.075361 | 72.965231 | Vouchered at the California Academy of Sciences (CAS237306); 12 dorsal spines and short snout |
| *Heniochus diphreutes* | Heniochus_diphreutes2b | RS7688 | October 27, 2014 | Farasan Islands, Saudi Arabia | 16.5767 | 42.23965 | 12 dorsal spines and short snout |
| *Heniochus intermedius* | Heniochus_intermedius | RS5740 | October 4, 2013 | Al Wajh, Saudi Arabia | 25.36156 | 36.912567 | Vouchered at the California Academy of Sciences (CAS236843) |
| *Istiophorus platypterus* | Istiophorus_platypterus | n/a | n/a | n/a | n/a | n/a | Outgroup, data obtained from Harrington et al. (2016) |
| *Mene maculatus* | Mene_maculatus | n/a | n/a | n/a | n/a | n/a | Outgroup, data obtained from Harrington et al. (2016) |
| *Naso unicornis* | Naso_unicornis | MEA1159 | July 16, 2012 | n/a | n/a | n/a | Outgroup, purchased from Quality Marine |
| *Platax orbicularis* | Platax_orbicularis1 | RS4764 | August 27, 2013 | Thuwal, Saudi Arabia | 22.266064 | 38.963594 | Outgroup |
| *Platax orbicularis* | Platax_orbicularis2 | RS5563 | September 30, 2013 | Gulf of Aqaba, Saudi Arabia | 28.40387 | 34.74067 | Outgroup; Vouchered at the California Academy of Sciences (CAS236791) |
| *Pomacanthus paru* | Pomacanthus_paru | n/a | n/a | n/a | n/a | n/a | Outgroup |
| *Prognathodes aculeatus* | Prognathodes_aculeatus | PAU01 | 2013 | Curaçao | n/a | n/a |  |
| *Prognathodes marcellae* | Prognathodes_marcellae | PMR06 | 2006 | Sao Tome and Principe | n/a | n/a |  |
| *Zanclus cornutus* | Zanclus_cornutus | ZeiCor1 | June, 2013 | Oahu, USA | n/a | n/a | Outgroup |
