# Supplementary material for: Ice ages and butterflyfishes: Phylogenomics elucidates the ecological and evolutionary history of reef fishes in an endemism hotspot
Source: Ecol Evol. 2018 Oct 23;8(22):10989–1008. doi: 10.1002/ece3.4566 (PMC6262737; doi:10.1002/ece3.4566)
Supplement: Supplementary file 5 [file ECE3-8-10989-s005.docx]

**Table S2.**  Biological, ecological, behavioral, and reproductive traits that were tested for their association with regional endemism

(endemic taxa indicated by asterisks). Traits include: dietary reliance on coral and coral reefs (V = very high – obligate corallivore, H

= high – facultative corallivore, M = moderate – benthic invertivore, L = low – planktivore; Cole & Pratchett 2013); habitat (C =

coral, R = rocky, D = deep reef, S = sediment, R = rubble, CO = coastal, CA = coastal algae); maximum depth (Allen *et al.* 1998);

social structure (S = single, P = paired, G = groups; Allen *et al.* 1998; Kuiter 2002; Yabutu & Berumen 2013); maximum body size

(TL in cm; Allen *et al.* 1998; Kuiter 2002).

| Species | Dietary reliance on coral reefs | Habitat | Maximum depth (m) | Social structure | Maximum body size (TL in cm) | Phylogenetic age (Ma) |
| --- | --- | --- | --- | --- | --- | --- |
|  |  |  |  |  |  |  |
| *Chaetodon auriga* | H | C | 40 | P | 23 | 2.7 |
| *Chaetodon auripes* | H | R, C, CA | 30 | P | 20 | 3 |
| *Chaetodon austriacus** | V | C | 20 | P | 13 | 1.3 |
| *Chaetodon baronessa* | V | C | 10 | P | 16 | 1.1 |
| *Chaetodon bennetti* | V | C | 30 | S | 20 | 3.5 |
| *Chaetodon collare* | H | C | 20 | P | 18 | 3.5 |
| *Chaetodon decussatus* | H | R, C | 30 | P | 20 | 1.8 |
| *Chaetodon dialeucos** | M | R | 25 | P | 18 | 2.2 |
| *Chaetodon falcula* | H | C | 15 | P | 20 | 2.4 |
| *Chaetodon fasciatus** | H | C | 25 | P | 22 | 1.9 |
| *Chaetodon gardineri** | H | CO | 40 | P | 17 | 1.4 |
| *Chaetodon guttatissimus* | V | C, S RU | 30 | P | 12 | 2.1 |
| *Chaetodon kleinii* | H | R, C | 61 | P | 15 | 1.3 |
| *Chaetodon larvatus** | V | C | 12 | P | 12 | 3.3 |
| *Chaetodon leucopleura* | H | C, S, RU | 80 | S | 17 | 1.4 |
| *Chaetodon lineolatus* | M | D, C | 171 | P | 30 | 2.9 |
| *Chaetodon lunula* | H | R, C | 30 | P | 20 | 1.9 |
| *Chaetodon lunulatus* | V | C | 20 | P | 14 | 1.7 |
| *Chaetodon madagaskariensis* | M | C | 120 | P | 13 | 1.8 |
| *Chaetodon melannotus* | V | C | 20 | S | 18 | 5 |
| *Chaetodon melapterus** | V | C, S | 16 | P | 13 | 1.3 |
| *Chaetodon mertensii* | H | R, C | 120 | P | 12.5 | 1.7 |
| *Chaetodon mesoleucos** | H | C | 20 | P | 13 | 1.7 |
| *Chaetodon nigropunctatus** | H | R, C | 18 | P | 14 | 1.7 |
| *Chaetodon oxycephalus* | M | C | 40 | P | 25 | 2.4 |
| *Chaetodon paucifasciatus** | H | RU, C | 30 | P | 14 | 1.6 |
| *Chaetodon pelewensis* | V | C | 30 | P | 12.5 | 1.8 |
| *Chaetodon pictus** | M | R, C | 20 | P | 20 | 1.8 |
| *Chaetodon plebeius* | V | C | 10 | S | 15 | 3.8 |
| *Chaetodon punctatofasciatus* | V | C | 45 | P | 12 | 1.8 |
| *Chaetodon semilarvatus** | V | C | 20 | P | 23 | 3.3 |
| *Chaetodon speculum* | H | C | 30 | S | 18 | 2 |
| *Chaetodon triangulum* | V | C | 15 | P | 16 | 1.1 |
| *Chaetodon trichrous* | H | C | 25 | P | 12 | 1.3 |
| *Chaetodon trifascialis* | V | C | 30 | S | 18 | 7.2 |
| *Chaetodon trifasciatus* | V | C | 20 | P | 15 | 2.5 |
| *Chaetodon unimaculatus* | V | C | 60 | P | 20 | 3.3 |
| *Chaetodon vagabundus* | M | C | 30 | P | 23 | 2.2 |
| *Chaetodon xanthurus* | H | C | 50 | P | 14 | 1.8 |
| *Chaetodon zanzibariensis* | V | C | 40 | P | 12 | 2 |
| *Forcipiger flavissimus* | M | C | 114 | P | 22 | 4.8 |
| *Forcipiger longirostris* | M | C | 60 | P | 22 | 4.8 |
| *Heniochus acuminatus* | M | CO, D | 75 | P | 25 | 2.5 |
| *Heniochus diphreutes* | L | D | 120 | G | 21 | 1.7 |
| *Heniochus intermedius** | H | C | 50 | P | 18 | 1.7 |

References:

Allen GR, Steene R, Allen M (1998) A Guide to Angelfishes and Butterflyfishes. Odyssey Publishing, Vanguard Press, Perth, WA.

Cole AJ, Pratchett MS (2013) Diversity in diet and feeding behaviour of butterflyfishes; reliance on reef corals versus reef habitats. Biology of

Butterflyfishes, 107.

Kuiter RH (2002) Butterflyfishes, Bannerfishes and their Relatives: A Comprehensive Guide to Chaetodontidae and Microcanthidae. TMC Publishing,

Chorleywood, UK.

Yabuta S, Berumen ML (2013) Social structures and spawning behaviour of *Chaetodon* butterflyfishes. Biology of Butterflyfishes, 200-225.
