# Supplementary material for: Ice ages and butterflyfishes: Phylogenomics elucidates the ecological and evolutionary history of reef fishes in an endemism hotspot
Source: Ecol Evol. 2018 Oct 23;8(22):10989–1008. doi: 10.1002/ece3.4566 (PMC6262737; doi:10.1002/ece3.4566)
Supplement: Supplementary file 6 [file ECE3-8-10989-s006.docx]

**Table S3.** Number of sequence reads per sample, assembled contigs, and UCE loci.

| Species | UCE_ID | Tissue ID | Reads (in millions) | Contigs | UCE loci |
| --- | --- | --- | --- | --- | --- |
| *Acanthurus olivaceus* | Acanthurus_olivaceus | MEA878 | 0.51 | 1629 | 998 |
| *Bothus pantherinus* | Bothus_pantherinus | Data obtained from Harrington et al. (2016) | | | |
| *Chaetodon auriga* | Chaetodon_auriga1 | RS5441 | 1.68 | 12450 | 962 |
| *Chaetodon auriga* | Chaetodon_auriga2 | RS5915 | 0.71 | 6022 | 948 |
| *Chaetodon auripes* | Chaetodon_auripes | 19 | 3.52 | 63552 | 955 |
| *Chaetodon austriacus* | Chaetodon_austriacus1 | RS5334 | 1.39 | 17528 | 700 |
| *Chaetodon austriacus* | Chaetodon_austriacus2 | RS3553 | 1.12 | 12342 | 940 |
| *Chaetodon baronessa* | Chaetodon_baronessa | 215088 | 1.3 | 15139 | 961 |
| *Chaetodon bennetti* | Chaetodon_bennetti1 | 3A | 1.89 | 23256 | 960 |
| *Chaetodon bennetti* | Chaetodon_bennetti2 | RS5997 | 1.37 | 14874 | 984 |
| *Chaetodon collare* | Chaetodon_collare1a | RS7171 | 1.36 | 16275 | 973 |
| *Chaetodon collare* | Chaetodon_collare1 | RS4767 | 1.85 | 2880 | 954 |
| *Chaetodon collare* | Chaetodon_collare2 | RS6035 | 1.16 | 9314 | 958 |
| *Chaetodon decussatus* | Chaetodon_decussatus | CDE100 | 1.29 | 19970 | 961 |
| *Chaetodon dialeucos* | Chaetodon_dialeucos1 | RS4179 | 1.72 | 16530 | 972 |
| *Chaetodon dialeucos* | Chaetodon_dialeucos2 | RS4509 | 1.32 | 17714 | 944 |
| *Chaetodon falcula* | Chaetodon_falcula | Cha_131 | 1.5 | 19462 | 958 |
| *Chaetodon fasciatus* | Chaetodon_fasciatus1 | RS5275 | 1.29 | 22771 | 934 |
| *Chaetodon fasciatus* | Chaetodon_fasciatus2 | RS1586 | 1.7 | 9213 | 953 |
| *Chaetodon cf. gardineri* | Chaetodon_gardineri | RS6242 | 1.31 | 13746 | 944 |
| *Chaetodon guttatissimus* | Chaetodon_guttatissimus1a | Cha_006 | 0.94 | 2370 | 920 |
| *Chaetodon guttatissimus* | Chaetodon_guttatissimus2b | RS6101 | 0.39 | 171 | 69 |
| *Chaetodon guttatissimus* | Chaetodon_guttatissimus3c | RS7049 | 4.85 | 9198 | 926 |
| *Chaetodon guttatissimus* | Chaetodon_guttatissimus4d | Cgut55 | 1.43 | 2417 | 818 |
| *Chaetodon guttatissimus* | Chaetodon_guttatissimus1 | Cha_040 | 1.46 | 805 | 364 |
| *Chaetodon interruptus* | Chaetodon_interruptus1a | M1456 | 1.17 | 11451 | 915 |
| *Chaetodon kleinii* | Chaetodon_kleinii1a | RS6944 | 0.64 | 1037 | 462 |
| *Chaetodon kleinii* | Chaetodon_kleinii2b | RS6997 | 1.9 | 15106 | 966 |
| *Chaetodon kleinii* | Chaetodon_kleinii3c | LAR2147 | 0.61 | 2916 | 948 |
| *Chaetodon kleinii* | Chaetodon_kleinii1 | Cha_046 | 1.24 | 968 | 517 |
| *Chaetodon kleinii* | Chaetodon_kleinii2 | RS5998 | 1.52 | 16867 | 955 |
| *Chaetodon larvatus* | Chaetodon_larvatus1 | RS1373 | 1.49 | 13212 | 951 |
| *Chaetodon larvatus* | Chaetodon_larvatus2 | RS1555 | 1.35 | 18949 | 895 |
| *Chaetodon leucopleura* | Chaetodon_leucopleura1 | RS4197 | 1.73 | 11855 | 914 |
| *Chaetodon leucopleura* | Chaetodon_leucopleura2 | RS6251 | 1.18 | 16235 | 948 |
| *Chaetodon lineolatus* | Chaetodon_lineolatus1a | RS7266 | 0.35 | 723 | 369 |
| *Chaetodon lineolatus* | Chaetodon_lineolatus1 | RS3822 | 1.29 | 7247 | 966 |
| *Chaetodon lineolatus* | Chaetodon_lineolatus2 | RS6277 | 0.66 | 38142 | 941 |
| *Chaetodon lunula* | Chaetodon_lunula1a | RS7283 | 0.75 | 1989 | 620 |
| *Chaetodon lunula* | Chaetodon_lunula1 | RS5960 | 2.51 | 9681 | 936 |
| *Chaetodon lunula* | Chaetodon_lunula2 | Cln1 | 0.98 | 9481 | 954 |
| *Chaetodon lunulatus* | Chaetodon_lunulatus | Clu394 | 1.02 | 16679 | 938 |
| *Chaetodon madagaskariensis* | Chaetodon_madagaskariensis | Cma11 | 1.6 | 9402 | 954 |
| *Chaetodon melannotus* | Chaetodon_melannotus1a | PI_049 | 3.6 | 17824 | 938 |
| *Chaetodon melannotus* | Chaetodon_melannotus1b | PI_307 | 2.09 | 16250 | 943 |
| *Chaetodon melannotus* | Chaetodon_melannotus1 | RS5467 | 0.96 | 11727 | 953 |
| *Chaetodon melannotus* | Chaetodon_melannotus2 | RS6189 | 1.44 | 8283 | 856 |
| *Chaetodon melapterus* | Chaetodon_melapterus1 | RS1541 | 1.43 | 11799 | 962 |
| *Chaetodon melapterus* | Chaetodon_melapterus2 | RS4650 | 1.52 | 1533 | 707 |
| *Chaetodon mertensii* | Chaetodon_mertensii | MCE_0385 | 1.16 | 5272 | 984 |
| *Chaetodon mesoleucos* | Chaetodon_mesoleucos1 | RS1451 | 0.93 | 5293 | 1004 |
| *Chaetodon mesoleucos* | Chaetodon_mesoleucos2 | RS1570 | 1.09 | 5322 | 974 |
| *Chaetodon nigropunctatus* | Chaetodon_nigropunctatus | RS4484 | 0.72 | 16202 | 956 |
| *Chaetodon nigropunctatus* | Chaetodon_nigropunctatus1a | RS4568 | 0.81 | 13019 | 955 |
| *Chaetodon nigropunctatus* | Chaetodon_nigropunctatus2b | RS4569 | 2.7 | 13883 | 950 |
| *Chaetodon nigropunctatus* | Chaetodon_nigropunctatus3c | RS4570 | 2.57 | 2062 | 859 |
| *Chaetodon oxycephalus* | Chaetodon_oxycephalus1a | RS6941 | 0.76 | 12627 | 945 |
| *Chaetodon paucifasciatus* | Chaetodon_paucifasciatus1 | RS5309 | 1.52 | 46185 | 952 |
| *Chaetodon paucifasciatus* | Chaetodon_paucifasciatus2 | RS3591 | 1.27 | 17120 | 955 |
| *Chaetodon pelewensis* | Chaetodon_pelewensis | Cpel2 | 1.47 | 8353 | 965 |
| *Chaetodon pictus* | Chaetodon_pictus1a | RS4121 | 0.75 | 2172 | 766 |
| *Chaetodon pictus* | Chaetodon_pictus1b | RS5922 | 1.68 | 1032 | 487 |
| *Chaetodon pictus* | Chaetodon_pictus1 | RS1567 | 1.37 | 3996 | 989 |
| *Chaetodon pictus* | Chaetodon_pictus2 | RS4814 | 1.17 | 12415 | 966 |
| *Chaetodon plebeius* | Chaetodon_plebeius | 215086 | 2.58 | 7648 | 998 |
| *Chaetodon punctatofasciatus* | Chaetodon_punctatofasciatus1a | PI_611 | 1.96 | 2238 | 860 |
| *Chaetodon semilarvatus* | Chaetodon_semilarvatus1 | RS5326 | 1.37 | 10954 | 977 |
| *Chaetodon semilarvatus* | Chaetodon_semilarvatus2 | RS1579 | 0.83 | 12511 | 853 |
| *Chaetodon speculum* | Chaetodon_speculum1a | PI_261 | 0.65 | 5047 | 990 |
| *Chaetodon speculum* | Chaetodon_speculum2b | PI_636 | 0.41 | 2146 | 831 |
| *Chaetodon triangulum* | Chaetodon_triangulum1a | RS6893 | 1.83 | 32138 | 961 |
| *Chaetodon triangulum* | Chaetodon_triangulum2b | RS6945 | 1.29 | 13672 | 938 |
| *Chaetodon trichrous* | Chaetodon_trichrous | Ctrich1 | 1.17 | 3602 | 992 |
| *Chaetodon trifascialis* | Chaetodon_trifascialis1a | RS7173 | 0.98 | 15014 | 968 |
| *Chaetodon trifascialis* | Chaetodon_trifascialis1 | RS5354 | 0.71 | 2635 | 930 |
| *Chaetodon trifascialis* | Chaetodon_trifascialis2 | Cha_044 | 1.46 | 8442 | 974 |
| *Chaetodon trifasciatus* | Chaetodon_trifasciatus | Cti5 | 1.22 | 24323 | 962 |
| *Chaetodon ulietensis* | Chaetodon_ulietensis1a | M1639 | 0.08 | 5963 | 987 |
| *Chaetodon unimaculatus* | Chaetodon_unimaculatus | NW13_245 | 1.96 | 25010 | 987 |
| *Chaetodon unimaculatus* | Chaetodon_unimaculatus1a | LAR 2142 | 4.65 | 17298 | 975 |
| *Chaetodon unimaculatus* | Chaetodon_unimaculatus2b | LAR 2143 | 1.89 | 4141 | 994 |
| *Chaetodon vagabundus* | Chaetodon_vagabundus1a | PI_310 | 1.64 | 9358 | 971 |
| *Chaetodon vagabundus* | Chaetodon_vagabundus2b | PI_635 | 0.33 | 28438 | 955 |
| *Chaetodon vagabundus* | Chaetodon_vagabundus1 | Cva3 | 1.33 | 4867 | 997 |
| *Chaetodon vagabundus* | Chaetodon_vaganbundus2 | 215096 | 1.37 | 2431 | 884 |
| *Chaetodon xanthurus* | Chaetodon_xanthurus1a | PI_048 | 1.25 | 3907 | 978 |
| *Chaetodon xanthurus* | Chaetodon_xanthurus2b | PI_156 | 3.92 | 6329 | 980 |
| *Chaetodon zanzibariensis* | Chaetodon_zanzibariensis1 | Cha_162 | 2.36 | 9980 | 957 |
| *Chaetodon zanzibariensis* | Chaetodon_zanzibariensis2 | RS6027 | 2.21 | 60624 | 977 |
| *Forcipiger flavissimus* | Forcipiger_flavissimus1a | RS7041 | 2.12 | 11593 | 948 |
| *Forcipiger flavissimus* | Forcipiger_flavissimus2b | XMAS_51 | 4.15 | 10361 | 943 |
| *Forcipiger flavissimus* | Forcipiger_flavissimus3c | XMAS_52 | 1.37 | 27814 | 992 |
| *Forcipiger flavissimus* | Forcipiger_flavissimus1 | NW13_439 | 1.9 | 11231 | 919 |
| *Forcipiger flavissimus* | Forcipiger_flavissimus2 | RS6140 | 1.8 | 11366 | 888 |
| *Forcipiger longirostris* | Forcipiger_longirostris | FOL2 | 2 | 17058 | 916 |
| *Forcipiger longirostris* | Forcipiger_longirostris1a | XMAS_50 | 2.95 | 1629 | 998 |
| *Heniochus acuminatus* | Heniochus_acuminatus2b | PI_123 | 3.27 | 12450 | 962 |
| *Heniochus acuminatus* | Heniochus_acuminatus | RS6013 | 0.61 | 6022 | 948 |
| *Heniochus diphreutes* | Heniochus_diphreutes1a | RS7394 | 1.04 | 63552 | 955 |
| *Heniochus diphreutes* | Heniochus_diphreutes2b | RS7688 | 2.14 | 17528 | 700 |
| *Heniochus intermedius* | Heniochus_intermedius | RS5740 | 0.96 | 12342 | 940 |
| *Istiophorus platypterus* | Istiophorus_platypterus | Data obtained from Harrington et al. (2016) | | | |
| *Mene maculatus* | Mene_maculatus | Data obtained from Harrington et al. (2016) | | | |
| *Naso unicornis* | Naso_unicornis | MEA1159 | 0.81 | 15139 | 961 |
| *Platax orbicularis* | Platax_orbicularis1 | RS4764 | 0.94 | 23256 | 960 |
| *Platax orbicularis* | Platax_orbicularis2 | RS5563 | 1.03 | 14874 | 984 |
| *Pomacanthus paru* | Pomacanthus_paru | n/a | 2.14 | 16275 | 973 |
| *Prognathodes aculeatus* | Prognathodes_aculeatus | PAU01 | 1.22 | 2880 | 954 |
| *Prognathodes marcellae* | Prognathodes_marcellae | PMR06 | 1.46 | 9314 | 958 |
| *Zanclus cornutus* | Zanclus_cornutus | ZeiCor1 | 1.99 | 19970 | 961 |
