# Supplementary material for: Ice ages and butterflyfishes: Phylogenomics elucidates the ecological and evolutionary history of reef fishes in an endemism hotspot
Source: Ecol Evol. 2018 Oct 23;8(22):10989–1008. doi: 10.1002/ece3.4566 (PMC6262737; doi:10.1002/ece3.4566)
Supplement: Supplementary file 7 [file ECE3-8-10989-s007.docx]

**Table S4.**  Model selection for the full phylogenetic linear multi-regression model obtained by dropping each variable independently in a backward stepwise manner (phylostep). We have shown the resulting Akaike information criterion (AIC). The selected models on each step are indicated in bold and the asterisk (*) indicates the final best model (with the lowest AIC score).

| Models | AIC  (*k* = 2) |
| --- | --- |
| *Starting model* |  |
| endemism ~ 1 + size + depth range + social + reliance + habitat + age | 82.12 |
|  |  |
| *Step 1* |  |
| endemism ~ 1 + size + social + reliance + habitat + age | 87.41 |
| endemism ~ 1 + size + depth range + social + reliance + habitat | 82.38 |
| endemism ~ 1 + size + depth range + reliance + habitat + age | 81.59 |
| endemism ~ 1 + size + depth range + social + habitat + age | 81.33 |
| endemism ~ 1 + size + depth range + social + reliance + age | 81.17 |
| **endemism ~ 1 + depth range + social + reliance + habitat + age** | **81.12** |
|  |  |
| *Step 2* |  |
| endemism ~ 1 + social + reliance + habitat + age | 85.99 |
| endemism ~ 1 + depth range + social + reliance + habitat | 80.57 |
| endemism ~ 1 + depth range + reliance + habitat + age | 79.65 |
| endemism ~ 1 + depth range + social + habitat + age | 79.38 |
| **endemism ~ 1 + depth range + social + reliance + age** | **79.17** |
|  |  |
| *Step 3* |  |
| endemism ~ 1 + social + reliance + age | 84.73 |
| endemism ~ 1 + depth range + social + reliance | 78.57 |
| endemism ~ 1 + depth range + reliance + age | 77.88 |
| **endemism ~ 1 + depth range + social + age** | **77.41** |
|  |  |
| *Step 4* |  |
| endemism ~ 1 + social + age | 85.66 |
| endemism ~ 1 + depth range + social | 76.66 |
| **endemism ~ 1 + depth range + age (*)** | **75.85** |
|  |  |
| *Step 5* |  |
| endemism ~ 1 + depth range | 76.00 |
