# Supplementary material for: Ice ages and butterflyfishes: Phylogenomics elucidates the ecological and evolutionary history of reef fishes in an endemism hotspot
Source: Ecol Evol. 2018 Oct 23;8(22):10989–1008. doi: 10.1002/ece3.4566 (PMC6262737; doi:10.1002/ece3.4566)
Supplement: Supplementary file 8 [file ECE3-8-10989-s008.docx]

**Outgroups to focal taxa.** Our outgroup sequence builds upon those previously used (Harrington *et al.* 2016) to include more proximal acanthomorph outgroups to chaetodontids and their immediate relatives. These additions derive from node-based calibrations in Harrington *et al.* (2016); justifications of minimum ages and phylogenetic placements are given there. The sequence of outgroups within acanthomorphs for this analysis includes: *Eospinus daniltsheknoi* as a representative of Tetraodontiformes (54.17 Ma. NB: This is a conservative marker relative to the controversial ‘plectocretacicoids’ and, in terms of resolution at the level of calcareous plankton zones, is coeval with the beaked tetraodontiform *Balkaria* [Bannikov *et al.* 2016]; age argumentation provided in Alfaro *et al.* in review); *Mene purdyi* as a representative of Carangimorpha (55.20 Ma); *Gasterorhamphosus zuppichini* as a representative of Syngnathiformes (69.71 Ma); *Stichocentrus liratus* as a representative of Holocentroidei (98.0 Ma); *Aipichthys minor* as a representative of Lampridiformes (98.0 Ma). These were then linked with the sequence of outgroups to acanthomorphs given by Harrington *et al.* (2016), which we repeat here for completeness. Aulopiformes (125 Ma, based on *Atolvorator longipectoralis*; see argumentation in Friedman *et al.* [2013]); non-eurypterygian Euteleostei (*Leptolepides haerteisi*, 150.94 Ma; see argumentation in Friedman *et al.* [2013] and Benton *et al.* [2015]); Otocephala (*Tischlingerichthys viholi*, 150.94 Ma; see argumentation in Benton *et al.* [2015]); Elopomorpha (†*Anaethalion zapporum*, 151.2 Ma; see argumentation in Benton *et al.* [2015] and Dornburg *et al.* [2015]); †Ichthyodectiformes (166.1 Ma, based on †*Occithrissops willsoni*; see argumentation in Friedman *et al.* [2013]); †*Leptolepis coryphaenoides* (181.7 Ma; see argumentation in Friedman *et al.* [2013]); †*Dorsetichthys bechei* (193.81 Ma; see argumentation for †*Pholidophorus bechei* in Friedman *et al.* [2013]); †Pholidophoridae (221.0 Ma, based on †*Knerichthys bronni*; approximate age estimate for the top of the Carnian given by Ogg [2012]); †*Prohalecites porroi* (236.0; based on age estimate and associated uncertainty for the top of the Norian given by Ogg [2012]); Holostei (247.1 Ma, based on †*Watsonulus eugnathoides*; see argumentation in Friedman *et al.* [2013]). As in Friedman *et al.* (2013), we specify a hard upper bound for our calculations of 322.8 Ma based on the stem neopterygian †*Discoserra*. This yields the following sequence of outgroup ages (in Ma): 247.1, 236.0, 221.0, 193.81, 181.7, 166.1, 151.2, 150.94, 150.94, 125, 98.0, 98.0, 69.71, 55.20, 54.17.

**Focal taxa to ingroup**

**Calibration 1**

**Node calibrated.** MRCA *Naso unicornis* and *Platax orbicularis*.

**Fossil taxon and specimen.** *Luvarus necopinatus*, PIN 2179/59, Borisyak Paleontological Institute, Russian Academy of Sciences, Moscow, Russia (Bannikov & Tyler 1995).

**Minimum age.** 54.17 Ma.

**Phylogenetic justification.** Bannikov & Tyler (1995) present an extensive verbal argument for placement of *Luvarus necopinatus* as a luvarid, and map synapmorphies onto a manually constructed phylogenetic hypothesis. Derived features supporting this placement are found throughout the skeleton and include, among others: presence of a massive pterygial truss, absence of epineurals, shortened 10^th^ caudal centrum, fusion of hypurals 1-4 to form a consolidated hypural plate, extreme hypurostegy, and consolidation of the pelvic fins into an operculum ani. The phylogenetic placement of luvarids as the sister group of a clade comprising zanclids and acanthurids is well supported by anatomical (Tyler *et al.* 1989) and molecular (Near *et al.* 2013) analyses.

**Age justification.** The Danatinsk Formation of Turkmenistan has been correlated with widely distributed sapropels associated with the Paleocene-Eocene boundary in the Peri-Tethys. This deposit spans calcareous nannoplankton zones NP9-NP10 (Gavriolv *et al.* 2003), yielding a minimum age of 54.17 Ma (Anthonissen & Ogg 2012).

**Estimated constraints on node-age prior.** 95% CI: 70.84 Ma.

**Outgroup age sequence.** 247.1, 236.0, 221.0, 193.81, 181.7, 166.1, 151.2, 150.94, 150.94, 125, 98.0, 98.0, 69.71, 55.20, 54.17, 54.17.

**Calibration 2**

**Node calibrated.** MRCA *Zanclus cornutus* and *Acanthurus olivaceus*.

**Fossil taxon and specimen.** *Eozanclus brevirostris*, MNHN BOL 4, Museum national d’ Histoire naturelle, Paris, France (Blot & Voruz 1975).

**Minimum age.** 49.0 Ma.

**Phylogenetic justification.** *Eozanclus* bears numerous derived features of Zanclidae outlined by Tyler et al. (1989), including: a single supranumerary spine on the first dorsal fin pterygiophore; an elevated count of anal and dorsal soft rays; a single predorsal bone; a triangular supraoccipital crest with a thickened anterior margin. More generally, *Eozanclus* also displays derived characters uniting Zanclidae and Acanthuridae (e.g. no pterygiophore inserting between neural spines of third and fourth vertebrae), as well as those uniting the two previous groups with Luvaridae (e.g. 9 + 13 vertebrae, scales bearing upright spinules [Tyler et al. 1989]).

**Age justification.** *Eozanclus brevirostris* is known from the Pesciara locality of Bolca, Italy. A detailed review of the geology and age of this deposit is given by Papazzoni *et al.* (2014), but key details are summarized here. Pesciara can be constrained to the narrow interval of overlap between NP14 and SBZ11. This constrains the deposits to no younger than 49 Ma (Vandenberghe *et al.* 2012), which we apply as a minimum age here.

**Estimated constraints on node-age prior.** 95% CI: 62.70 Ma.

**Outgroup age sequence.** 247.1, 236.0, 221.0, 193.81, 181.7, 166.1, 151.2, 150.94, 150.94, 125, 98.0, 98.0, 69.71, 55.20, 54.17, 54.17, 49.0.

**Calibration 3**

**Node calibrated.** MRCA *Naso unicornis* and *Acanthurus olivaceus*.

**Fossil taxon and specimen.** *Proacanthurus tenuis*, MCSNV I.G.23694, Museo Civico di Storia Naturale, Verona, Italy (Sorbini & Tyler 1998).

**Minimum age.** 49.0 Ma.

**Phylogenetic justification.** *Proacanthurus* bears a folding spine in the caudal peduncle. This feature is considered derived relative to the fixed, plate-like caudal armature found in nasines and the acanthurine *Prionurus* (Tyler *et al.* 1989; Sorbini & Tyler 1998). The presence of such a spine in *Proacanthurus* aligns the genus with the subset of nasines showing this derived morphology, which collectively appear to be the monophyletic sister lineage of *Prionurus* (Ludt *et al.* 2015).

**Age justification.** *Proacanthurus tenuis* is known from the Pesciara locality of Bolca, Italy. A detailed review of the geology and age of this deposit is given by Papazzoni *et al.* (2014), but key details are summarized here. Pesciara can be constrained to the narrow interval of overlap between NP14 and SBZ11. This constrains the deposits to no younger than 49 Ma (Vandenberghe *et al.* 2012), which we apply as a minimum age here.

**Estimated constraints on node-age prior.** 95% CI: 57.22 Ma.

**Outgroup age sequence.** 247.1, 236.0, 221.0, 193.81, 181.7, 166.1, 151.2, 150.94, 150.94, 125, 98.0, 98.0, 69.71, 55.20, 54.17, 54.17, 49.0, 49.0.

**Calibration 4**

**Node calibrated.** MRCA of *Pomacanthus paru* and *Chaetodon ocellatus*.

**Fossil taxon and specimen.** Chaetodontidae indet. (tholichthys larval stage)., HLMD T-410a and counterpart HLMD WT-410b, Hessisches Landesmuseum, Darmstadt, Germany (Micklich *et al.* 2009).

**Minimum age.** 29.62 Ma.

**Phylogenetic justification.** The specimen bears distinctive features of tholichthys-stage larvae of chaetodontids: plate-like posterior extensions of the supracleithrum and posttemporal, an expanded preoperculum bearing a posterior spine, and a rugose surface texture to many skull bones (Micklich *et al.* 2009).

**Age justification.** The ‘fish shales’ of Grube Unterfeld (“Frauenweiler”) yielding Echenidae undet. lie within NP23 (Sakamoto *et al.* 2004). The top of NP23 is dated to 29.62 Ma (Vandenberghe *et al.* 2012), providing a minimum age of divergence between *Pomacanthus paru* and *Chaetodon ocellatus*.

**Estimated constraints on node-age prior.** 95% CI: 59.26 Ma.

**Outgroup age sequence.** 247.1, 236.0, 221.0, 193.81, 181.7, 166.1, 151.2, 150.94, 150.94, 125, 98.0, 98.0, 69.71, 55.20, 54.17, 54.17, 49.0, 49.0, 29.62.

**Notes.** Two adult body fossils (rather than the tholichthys larvae discussed above) of putative chaetodontids are known from strata of late Oligocene age, but neither can be placed with certainty. The first of these is *Chaetodon hoeferi* from the Laško-Trbovlje syncline of Slovenia (Gorjanovic-Kramberger 1898). This fossil is often reported as Rupelian based on biostratigraphy (Carnevale 2006), but radiometric studies indicate a Chattian age (Bechtel *et al.* 2004). Illustrations of the fossil shows clear synapomorphies of Chaetodontidae (Blum 1988), including: apparent sequential articulation between the supraoccipital crest, supraneruals, and first dorsal-fin pterygiophore; long ribs; expanded flanges on ribs. In overall appearance, the fossil does resemble *Chaetodon* (Carnevale 2006), but ambiguities surrounding both the characters of individual chaetodontid lineages and the morphology of *Chaetodon hoeferi* prevent a more precise placement than Chaetodontidae. The second putative Oligocene chaetodontid body fossil is an incomplete specimen from Azerbaijan missing the skull. Named as *Chaetodon penniger* (Bogachev 1964), this taxon is in need of systematic revision (Bannikov & Parin 1997) and is thus unreliable as a calibration at present.

**Calibration 5**

**Node calibrated.** MRCA of *Prognathodes marcellae* and *Chaetodon ocellatus*.

**Fossil taxon and specimen.** *Chaetodon ficheuri*, MNHN ORA89, Saint-Denis du Sig, Algeria (Carnevale 2006).

**Minimum age.** 5.94 Ma.

**Phylogenetic justification.** Detailed redescription provides extensive discussion justifying placement of *Chaetodon ficheuri* within *Chaetodon* (Carnevale 2006). Insofar as they have been utilized as characters in cladistic analyses of chaetodontids and are easily observable in fossil material (Blum 1988; Smith *et al.* 2003), exclusion of the second circumorbital from the orbital margin and the presence of an incomplete supracleithral canal is the clearest evidence for this assignment. Within *Chaetodon* itself, placement of *C. ficheuri* is uncertain. Carnevale suggests it is more closely related to Indo-Pacific subgenera rather than the Atlantic subgenus *Chaetodon*. However, our analysis does not include any of the three species assigned to that subgenus, so instead we apply *C. ficheuri* as a minimum marker for the divergence between *Chaetodon* and *Prognathodes*.

**Estimated constraints on node-age prior.** The fish-bearing rocks near Oran, Algeria, date to the onset of diatomite sedimentation in the Chelif Basin, which is placed within the Messinian on the basis of planktonic foraminiferans (Mansour *et al.* 1995). As in other classic Messinian sequences, the Algerian diatomites lie beneath the evaporitic gypsum strata representing dessication of the Mediterranean basin. This geographically synchronous onset of the Messinian Salinity Crisis is dated to 5.96 ± 0.02 Ma by astrochronology (Krijgsman *et al.* 1999; Krijgsman *et al.* 2001), giving a minimum age of 5.94 Ma for *Chaetodon ficheuri*.

**Estimated constraints on node-age prior.** 95% CI: 47.50 Ma.

**Outgroup age sequence.** 247.1, 236.0, 221.0, 193.81, 181.7, 166.1, 151.2, 150.94, 150.94, 125, 98.0, 98.0, 69.71, 55.20, 54.17, 54.17, 29.62, 5.94.

**References**

Anthonissen DE, Ogg JG (2012) Cenozoic and Cretaceous biochronology of planktonic foraminifera and calcareous nannofossils. *The Geological Time Scale*. Gradstein FM, Ogg JG, Schmitz MD, Ogg GM. Amsterdam, Elsevier. **2,** 1083-1127.

Bannikov AF, Parin NN (1997) The list of marine fishes from Cenozoic (upper Paleocene-middle Miocene) localities in southern European Russia and adjacent countries. *Journal of Ichthyology* **37,** 133-146.

Bannikov AF, Tyler JC (1995) Phylogenetic revision of the fish families Luvaridae and †Kushlukiidae (Acanthuroidei), with a new genus and two new species of Eocene luvarids. *Smithsonian Contributions to Paleobiology* **81,** 1-45.

Bannikov AF et al. (2016) A new family of gymnodont fish (Tetraodontiformes) from the earliest Eocene of the Peri-Tethys (Kalbardino-Balkaria, northern Caucasus, Russia). *Journal of Systematic Palaeontology* **15,** 129-146.

Bechtel A et al. (2004) Paleoenvironment of the upper Oligocene Trbovlje coal seam (Slovenia). *International Journal of Coal Geology* **2004,** 23-48.

Benton MJD et al. (2015) Contraints on the timescale of animal evolutionary history. *Palaeontologia Electronica* **18.1.1FC,** 1-107.

Blot J, Voruz C (1975) La famille des Zanclidae. *Studi e Ricerche sui giacimenti terziari di Bolca* **2,** 233-271.

Blum SD (1988) The osteology and phylogeny of the Chaetodontidae (Teleostei: Perciformes), University of Hawaii. **PhD,** 365.

Bogachev VV (1964) Paleoichthyological notes." *Trudy Azerbaydzhanskogo Instituta Dobyche Nefti* **13,** 9-31.

Carnevale G (2006) Morphology and biology of the Miocene butterflyfish *Chaetodon ficheuri* (Teleostei: Chaetodontidae). *Zoological Journal of the Linnean Society* **146,** 251-267.

Dornburg A et al. (2015) Phylogenetic analysis of molecular and morphological data highlights uncertainty in the relationships of fossil and living species of Elopomorpha (Actinopterygii: Teleostei). *Molecular Phylogenetics and Evolution* **89,** 205-218.

Friedman M et al. (2013) Molecular and fossil evidence place the origin of cichlid fishes long after Gondwanan rifting. *Proceedings of the Royal Society B* **280,** 20131733.

Friedman M et al. (2013) Molecular and fossil evidence place the origin of cichlid fishes long after Gondwanan rifting. *Proceedings of the Royal Society B: Biological Sciences* **280,** 1770.

Gavriolv YO et al. (2003) Paleocene-Eocene boundary events in the northeaster Peri-Tethys. *Geological Society of America Special Paper* **369,** 147-168.

Gorjanovic-Kramberger D (1898) Über fossile Fische von Tüffer in Steiermark un Jurjevèani in Croatien. *Glasnika Hrvatsko Naravoslovnog Društva* **10,** 24-34.

Harrington RC et al. (2016) Phylogenomic analysis of carangimorph fishes reveals flatfish asymmetry arose in a blink of the evolutionary eye. *BMC Evolutionary Biology* **16,** 224.

Krijgsman W et al. (2001) Astrochronology for the Messinian Sorbas basin (SE Spain) and orbital (precessional) forcing for evaportie cyclicity. *Sedimentary Geology* **140,** 43-60.

Krijgsman W et al. (1999) Chronology, causes and progression of the Messinian Salinity Crisis. *Nature* **400,** 652-655.

Ludt WB et al. (2015) Skipping across the tropics: The evolutionary history of sawtail surgeonfishes (Acanthuridae: *Prionurus*). *Molecular Phylogenetics and Evolution* **84,** 166-172.

Mansour B et al. (1995) L'enregistrement par les associations de diatomées des environments messiniens: l'example de la coupe Sig (bassin Chélif-Algerie). *Geobios* **28,** 261-279.

Micklich NR et al. (2009) First records of the tholichthys larval stage of butterfly fishes (Perciformes, Chaetodontidae), from the Oligocene of Europe. *Paläontolgische Zeitschrift* **83,** 479-497.

Near TJ et al. (2013) Phylogeny and tempo of diversification in the superradiation of spiny-rayed fishes. *Proceedings of the National Academy of Sciences of the USA* **110,** 12738-12743.

Ogg JG (2012) Triassic. *The Geological Time Scale*. Gradstein FM, Ogg JG, Schmitz MD, Ogg GM. Amsterdam, Elsevier. 681-730.

Papazzoni CA et al. (2014) The Pesciara-Monte Postale *Fossil-Lagerstätte*: 1. Biostratography, sedimentology and despositional model. *Reconditi della Società Paleontologica Italiana* **4,** 29-36.

Sakamoto K et al. (2004) *Oligopleuronectes germanicus* gen. et sp. nov., an Oligocene pleuronectid flatfish from Fauenweiler, S-Germany. *Bulletin of the National Science Museum, Tokyo, Series C* **30,** 89-94.

Smith WL et al. (2003) The evolution of the laterophysic connection with a revised phylogeny and taxonomy of butterflyfishes (Teleostei: Chaetodontidae). *Cladistics* **19,** 287-306.

Sorbini L, Tyler JC (1998) A new species of the Eocene surgeon fish genus *Pesciaraichthys* from Monte Bolca, Italy (Acanthuridae), with comments on caudal peduncle armature and supraneurals in acanthurids. *Studi e Ricerche sui giacimenti terziari di Bolca* **7,** 21-34.

Tyler JC et al. (1989) Morphology of *Luvarus imperialis* (Luvaridae), with a phylogenetic analysis of the Acanthuroidei (Pisces). *Smithsonian Contributions to Zoology* **485,** 1-78.

Vandenberghe N et al. (2012) The Paleogene Period. *The Geologic Timescale*. Gradstein FM, Ogg JG, Schmitz MD, Ogg GM. Amsterdam, Elsevier. **2,** 855-921.
