# Supplementary material for: Ice ages and butterflyfishes: Phylogenomics elucidates the ecological and evolutionary history of reef fishes in an endemism hotspot
Source: Ecol Evol. 2018 Oct 23;8(22):10989–1008. doi: 10.1002/ece3.4566 (PMC6262737; doi:10.1002/ece3.4566)
Supplement: Supplementary file 9 [file ECE3-8-10989-s009.docx]

**Figure S1.** A fossil calibrated chronogram for all Chaetodontidae species included in this

study based on analysis of ultraconserved element (UCE) data. The time scale is calibrated in

millions of years before present, calibration nodes are indicated by red circles, and the

numbers within parentheses show the 95% credible interval for those nodes.
