# Supplementary material for: Ice ages and butterflyfishes: Phylogenomics elucidates the ecological and evolutionary history of reef fishes in an endemism hotspot
Source: Ecol Evol. 2018 Oct 23;8(22):10989–1008. doi: 10.1002/ece3.4566 (PMC6262737; doi:10.1002/ece3.4566)
Supplement: Supplementary file 10 [file ECE3-8-10989-s010.docx]

**Figure S2.** Clade by clade comparison of taxon sampling and geographic range occupancy in our UCE calibrated phylogeny and the published phylogeny of Cowman & Bellwood (2011) (CB here after). Presence-absence matrix displays species occupancy across broad geographic ranges. Colored bars at nodes indicate 95% highest posterior densities (HPD) or range estimates around reconstructed node aging. Bars are not shown for nodes that did not receive over 0.50 Bayesian posterior clade support in the CB reconstruction. Grey colored branches in the CB clades highlight extant lineages or potential lineages not sampled in the new reconstruction using UCE data; green indicates that the UCE study does not sample the sibling to that species. Conversely, orange colored lineages in the UCE clades represent species not previously sampled by CB. With the exception of the bannerfish clade, our current taxon sampling has the ability to capture the crown node of each subclade in the family Chaetodontidae. Overlap in the 95% HPD interval of clade crown nodes among studies are indicated by shaded boxes. In all clades considered here, the current sampling of UCE data for butterflyfish provides the most comprehensive phylogenetic resolution for Red Sea lineages and their closely related species to date.
