# Supplementary material for: Ice ages and butterflyfishes: Phylogenomics elucidates the ecological and evolutionary history of reef fishes in an endemism hotspot
Source: Ecol Evol. 2018 Oct 23;8(22):10989–1008. doi: 10.1002/ece3.4566 (PMC6262737; doi:10.1002/ece3.4566)
Supplement: Supplementary file 11 [file ECE3-8-10989-s011.docx]

**Figure S3.** Ages reconstructed from UCE data focusing on the origins of Red Sea resident

butterflyfishes when compared to the same species sampled in two previous studies that focus

on a broader phylogenetic reconstruction of the family Chaetodontidae; Cowman &

Bellwood (2011) in pink, Hodge *et al.* (2014) in green. Lines represent the 95% highest

posterior densities of the UCE data from the MCMCTree calibrated phylogeny presented in

Figure 2, as well as the published BEAST calibrated chronograms of Cowman & Bellwood

(2011) and Hodge *et al.* (2014). The inset box plot displays the distribution of median

divergence time estimates for Red Sea resident butterflyfish for all three chronograms.

Species marked by asterisks are considered to be regional endemics. Four Red Sea resident

butterflyfish species included in our UCE phylogeny (*Chaetodon leucopleura, C. melapterus,*

*C. pictus, Heniochus intermedius*) have not previously been sampled in this context.

Significant geological events in the coastal waters of the Arabian Peninsula are temporally

indicated by red dashed lines.
